# The Efficacy and Safety of Sodium Bicarbonate Ringer’s Solution in Critically Ill Patients: A Retrospective Cohort Study

**DOI:** 10.3389/fphar.2022.829394

**Published:** 2022-03-30

**Authors:** Yi Bian, Tingting Xu, Yue Le, Shusheng Li

**Affiliations:** ^1^ Department of Emergency Medicine, Tongji Hospital, Tongji Medical College, Huazhong University of Science and Technology, Wuhan, China; ^2^ Department of Critical Care Medicine, Tongji Hospital, Tongji Medical College, Huazhong University of Science and Technology, Wuhan, China

**Keywords:** sodium bicarbonate Ringer’s solution, saline, critically ill patients, outcome, safety

## Abstract

**Background:** Sodium bicarbonate Ringer’s solution has been widely used in clinical practice in recent years. There are few clinical studies on the efficacy and safety of this fluid among critically ill patients until now.

**Method:** This retrospective cohort study included critically ill adult patients in the intensive care unit (ICU) of Tongji Hospital from 1 January 2019 to 31 December 2020. By reviewing exclusively the use of either sodium bicarbonate Ringer’s solution or saline for resuscitation or maintenance, the patients were included into two groups, respectively. The primary outcome was the major adverse kidney event within 30 days (MAKE30), including death, new receipt of renal replacement therapy, or persistent renal dysfunction. Safety outcomes were focused on arterial blood gas and plasma biochemical alterations, which might potentially be induced by the administration of bicarbonate Ringer’s solution.

**Result:** A total of 662 patients were included in the cohort. Compared to the saline group, the bicarbonate Ringer’s group had a significantly lower rate of the new receipt of renal replacement therapy [adjusted odds ratio (OR) = 0.591, 95% confidence interval (CI), 0.406 to 0.861; *p* = 0.006]. There was no significant difference between the two groups in 30-day mortality, final creatinine level ≥200% of baseline, and major adverse kidney event within 30 days. In subgroup analysis, the incidence of MAKE30 was higher in the bicarbonate Ringer’s group than that of the saline group among patients with cardiovascular disease. The patients in the bicarbonate Ringer’s group had a longer length of intensive care unit stay than patients in the saline group, but their new renal replacement therapy days were shorter. No major alterations were found in arterial blood gas and plasma biochemical during the follow-up period.

**Conclusion:** Compared to saline, sodium bicarbonate Ringer’s solution exhibited a potential renal function protective effect while causing no major alterations in arterial blood gas and plasma biochemistry. However, the application in patients with cardiovascular disease diagnosis at ICU admission should be cautious.

## Introduction

In the intensive care unit (ICU), severe shock with hemodynamic instability is one of the most important pathophysiological changes endangering the life of critically ill patients. Fluid resuscitation is an important and effective treatment to improve tissue hypoperfusion (Gordon and Spiegel, 2020). Crystalloid fluid is recommended to use as the resuscitation fluid for septic shock in the Surviving Sepsis Campaign in 2021 (Evans et al., 2021). Crystalloid fluid mainly includes saline (0.9% sodium chloride) and balanced crystalloids (also known as Ringer’s solution).

Saline is the most commonly administered intravenous fluid (Finfer et al., 2010). Previous studies suggested that intravenous saline may be associated with hyperchloremic metabolic acidosis and acute kidney injury (Yunos et al., 2012; Semler and Rice, 2016). One of the recent randomized controlled trial studies, Isotonic Solutions and Major Adverse Renal Events Trial showed that the use of balanced crystalloids in critically ill patients may reduce the incidence of acute kidney injury, renal replacement therapy, and death (Barea-Mendoza et al., 2018). Therefore, the balanced crystalloids are supposed to be more conducive to clinical prognosis and bring benefits to patients, compared to saline.

At present, the most widely used balanced crystalloids include sodium lactate Ringer’s solution, sodium acetate Ringer’s solution, and the newly emerging sodium bicarbonate Ringer’s solution. Sodium lactate Ringer’s solution and sodium acetate Ringer’s solution contain lactate and acetate, respectively. Lactate and acetate are metabolized through a variety of tissues and organs, which may increase the burden of liver and kidney function (Shin et al., 2011; McCague et al., 2012). Sodium bicarbonate Ringer’s solution may have its unique advantages. Relevant animal experiments and phase I and phase II clinical studies have explained to a certain extent the pharmacological mechanism and pathophysiological effects of sodium bicarbonate Ringer’s solution on patients (Satoh et al., 2005a; Satoh et al., 2005b; Sasano et al., 2007; Pakfetrat et al., 2019). Sodium bicarbonate Ringer’s solution contains a physiological bicarbonate buffer system that can quickly exert the alkalinization effect without affecting the physiological metabolism to alleviate metabolic acidosis, restore the body’s self-regulation ability, and avoid iatrogenic alkalemia (Satoh et al., 2005a; Satoh et al., 2005b; Sasano et al., 2007; Pakfetrat et al., 2019).

At present, a large number of different brands of sodium bicarbonate Ringer’s solutions are used in clinics, but there is still a lack of clinical studies to observe its impact on the prognosis of patients and to verify its safety in the real world. To this end, we conducted this retrospective cohort study to explore the association between sodium bicarbonate Ringer’s solution and the prognosis of critically ill patients, as well as its clinical safety.

## Materials and Methods

### Ethical Considerations

This retrospective observational study was approved by the Medical Ethics Committee of Tongji Hospital, Tongji Medical College, Huazhong University of Science and Technology (No. TJ-IRB202100507). The clinical trial was registered and verified at the Chinese Clinical Trial Registry (ChiCTR2100050350).

### Study Design and Participants

This retrospective cohort study was conducted in the ICU of Tongji Hospital, Tongji Medical College, Huazhong University of Science and Technology in Wuhan, China. We collected the clinical data of patients either using sodium bicarbonate Ringer’s solution or saline for fluid resuscitation or maintenance treatment in the ICU from 1 January 2019 to 31 December 2020. As a retrospective study, all decisions of fluid management were made by bedside physicians, including the type, amount, and timing of fluids to be used. We screened the patients who met the inclusion criteria: 1) length of ICU stay ≥24 h and 2) use more than 500 ml of sodium bicarbonate Ringer’s solution or saline. The patients were excluded from the study if they 1) were <14 years old; 2) were a pregnant or lactating woman; 3) had hypermagnesemia or hypothyroidism; 4) were with insufficient clinical information; 5) used other types of crystal solutions beside saline and sodium bicarbonate Ringer’s solution, including but not limited to sodium lactate Ringer’s solution and sodium acetate Ringer’s solution; or 6) used both saline and sodium bicarbonate Ringer’s solution for fluid resuscitation or maintenance treatment during ICU stay.

### Data Collection

The patients’ characteristics and clinical information were obtained from the hospital’s electronic medical and nursing record system. Trained reviewers validated and expanded the data using standardized data collection forms. The clinical data included demographic characteristics, chronic comorbidities, physiological status at ICU admission, arterial blood gas, renal function, electrolytes, use of ventilators and vasopressors, new receipt of renal replacement therapy, and vital status at hospital discharge. Data of cumulative volume, administration time, duration of saline or sodium bicarbonate Ringer’s solution were collected. An amount of 500 ml fluid infusing within 1 h was defined as resuscitation, while that longer than 1 h was defined as maintenance.

### Prognostic Related Indicators and Data Measurement

The primary outcome was the incidence of major adverse kidney event within 30 days (MAKE30), which included death, new receipt of renal replacement therapy, or persistent renal dysfunction (defined as a final inpatient plasma creatinine value ≥200% of the baseline value)—all censored at hospital discharge or 30 days in the hospital, whichever came first (Kashani et al., 2013; Semler et al., 2016; Kellum et al., 2017; Semler et al., 2017; Barea-Mendoza et al., 2018). The following-up of the cohort began at the ICU admission (zero point) and lasted for 30 days. We regarded the most recent creatinine value before hospital admission or the creatinine value at hospital admission as the baseline value for renal function assessment, in which the former one was given priority over the later one. For patients with chronic kidney disease, the lowest creatinine value during hospitalization was regarded as the baseline value of creatinine. The patients who had a previous history of routine renal replacement therapy before enrollment cannot be considered to meet the criteria of new renal replacement therapy or persistent renal dysfunction, but could be eligible for the primary outcome if they died during hospital stay (Semler et al., 2017; Barea-Mendoza et al., 2018).

The secondary outcomes were the length of stay in the hospital, length of stay in the ICU, the length of vasopressors application, the length of new renal replacement therapy, the length of mechanical ventilation, and cumulative volume of saline or sodium bicarbonate Ringer’s solution for resuscitation or maintenance within the first 24 h of the ICU.

The safety outcome indicators mainly concern whether the results of arterial blood gas analysis and plasma biochemical indexes altered after the administration of saline or sodium bicarbonate Ringer’s solution and whether differences exist between groups. We collected the results of arterial blood gas analysis before and 6 and 24 h after the use of saline or sodium bicarbonate Ringer’s solution. We collected blood biochemical indexes (chloride, creatinine and bicarbonate) before and within 6 days after using saline or sodium bicarbonate Ringer’s solution. For these repeated measurement data, the results of arterial blood gas analysis and blood biochemical indexes before the use of saline or sodium bicarbonate Ringer’s solution were considered the baseline.

### Statistical Analysis

As this study was a retrospective observational study, data missing were inevitable. Variables at ICU admission with missing data greater than 20% were excluded from this analysis. Multiple imputations were conducted for addressing the presence of missing values at ICU admission. Repeated measurement data were reported with available numbers of patients.

Categorical variables were compared by Pearson χ^2^ test or Fisher’s exact test, as appropriate. Continuous variables were compared by the Mann–Whitney U test. Continuous variables were reported as medians and interquartile range (IQR). Categorical variables were reported as frequencies and proportions. The categorical outcomes were analyzed using the logistic regression model by adjusting the age, gender, admission source, sequential organ failure assessment (SOFA) score at ICU admission and diagnosis on ICU admission, including medical diseases, cardiovascular disease, neurological disease, traumatic, surgical, sepsis and traumatic brain injury. To address the linearity relationship between the categorical outcomes and covariates, binary logistic regression by the Box–Tidwell method was conducted (Cheng et al., 2021). Collinearity diagnostic analysis was done to clarify the potential multi-collinearity between covariates by evaluating the tolerance and variance inflation factor (VIF) value (Ripa et al., 2021). In the repeated measurement data analysis, we used two-way ANOVA for comparison between the groups as well as to detect change with time. Subgroup analyses were stratified by admission type, admission source, use of mechanical ventilation, use of vasopressors and diagnosis including sepsis, septic shock, traumatic brain injury and chronic kidney disease. Statistical analyses were performed using SPSS version 20.0 software (IBM Corp, Armonk, New York, United States), while GraphPad Prism version 5.0 software (GraphPad Software Inc., La Jolla, CA, United States) was used to construct the forest plot. A bilateral *p*-value of less than 0.05 was considered to be a statistically significant difference.

## Results

### Baseline Characteristics

**FIGURE 1:**
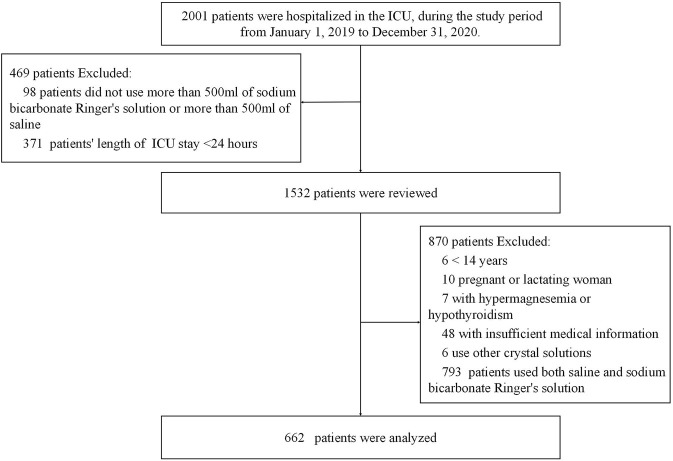
Flowchart. Abbreviations: ICU, intensive care unit.

The study finally included 662 patients in the cohort (Figure 1). The median age of patients was 54 years (IQR, 43–66), with 65.3% of the male gender. There were 270 patients in the bicarbonate Ringer’s group and 392 patients in the saline group. At ICU admission, there were no significant differences in demographic characteristics and major severity indicators including use of vasopressors, type of oxygen therapy and SOFA score (Table 1).

**TABLE 1:** Participants’ baseline characteristics at ICU admission.

Characteristic	Total (n = 662)	Saline (n = 392)	Bicarbonate Ringer’s (n = 270)	*p-*value
Male sex, no. (%)	432 (65.3)	260 (66.3)	172 (63.7)	0.486
Age, median (IQR), years	54 (43–66)	54 (43–66)	55 (43–66)	0.542
Chronic comorbidities				
Hypertension, no. (%)	183 (27.6)	109 (27.8)	74 (27.4)	0.910
Coronary atherosclerotic heart disease, no. (%)	45 (6.8)	29 (7.4)	16 (5.9)	0.460
Chronic obstructive pulmonary disease, no. (%)	13 (2.0)	8 (2.0)	5 (1.9)	0.863
Chronic liver disease, no. (%)	72 (10.9)	38 (9.7)	34 (12.6)	0.239
Diabetes, no. (%)	91 (13.7)	58 (14.8)	33 (12.2)	0.345
Tumor, no. (%)	59 (8.9)	29 (7.4)	30 (11.1)	0.099
Immunosuppressive state, no. (%)	31 (4.7)	12 (3.1)	19 (7.0)	0.017
Chronic kidney disease, no. (%)	47 (7.1)	26 (6.6)	21 (7.8)	0.573
Source of admission to ICU, no. (%)				
Emergency department	279 (42.1)	176 (44.9)	103 (38.1)	0.052
Hospital ward	346 (52.3)	200 (51.0)	146 (54.1)	
Others	37 (5.6)	16 (4.1)	21 (7.8)	
Diagnosis on ICU admission, no. (%)				
Medical diseases	539 (81.4)	321 (81.9)	218 (80.7)	0.709
Cardiovascular disease	139 (21.0)	65 (16.6)	74 (27.4)	0.001
Neurological disease	26 (3.9)	7 (1.8)	19 (7.0)	0.001
Traumatic	173 (26.1)	96 (24.5)	77 (28.5)	0.246
Surgical	289 (43.7)	170 (43.4)	119 (44.1)	0.857
Sepsis	170 (25.7)	109 (27.8)	61 (22.6)	0.131
Septic shock	137 (20.7)	87 (22.2)	50 (18.5)	0.251
Traumatic brain injury	81 (12.2)	46 (11.7)	35 (13.0)	0.636
Renal insufficiency	192 (29.0)	121 (30.9)	71 (26.3)	0.203
Consciousness of admission, no. (%)				
Consciousness	295 (44.6)	187 (47.7)	108 (40.0)	0.098
Somnolence	98 (14.8)	61 (15.6)	37 (13.7)	
Stupor	40 (6.0)	18 (4.6)	22 (8.1)	
Shallow coma	173 (26.1)	97 (24.7)	76 (28.1)	
Deep coma	56 (8.5)	29 (7.4)	27 (10.0)	
Vasopressors of admission, no. (%)	184 (27.8)	103 (26.3)	81 (30.0)	0.293
Oxygen therapy of admission, no. (%)				
Without oxygen inhalation	6 (0.9)	3 (0.8)	3 (1.1)	0.736
Nasal catheter oxygen inhalation	304 (45.9)	190 (48.5)	114 (42.2)	
Mask oxygen inhalation	95 (14.4)	53 (13.5)	42 (15.6)	
High flow nasal cannula therapy	6 (0.9)	3 (0.8)	3 (1.1)	
Non-invasive mechanical ventilation	7 (1.1)	4 (1.0)	3 (1.1)	
Invasive mechanical ventilation	244 (36.9)	139 (35.5)	105 (38.9)	
Physiological status of admission				
Heart rate (times/min)	100 (84–116)	101 (84–117)	98 (81–115)	0.420
Respiratory rate (times/min)	20 (18–24)	20 (17–24)	20 (18–23)	0.438
Pulse rate (times/min)	100 (84–116)	101 (84–117)	98 (81–115)	0.424
Systolic blood pressure (mmHg)	117 (96–137)	118 (97–138)	115 (95–136)	0.414
Diastolic blood pressure (mmHg)	67 (55–78)	67 (55–79)	65 (54–76)	0.083
Peripheral blood oxygen saturation (%)	100 (97–100)	100 (97–100)	100 (97–100)	0.376
Fraction of inspired oxygen (%)	41 (35–60)	41 (33–60)	41 (37–61)	0.031
Sequential organ failure assessment score of admission	6 (4–9)	6 (4–9)	6 (4–10)	0.521
Laboratory examination laboratory examination				
Potassium (mmol/L)	4.2 (3.8–4.6)	4.2 (3.8–4.6)	4.2 (3.8−4.6)	0.797
Sodium (mmol/L)	140.0 (136.3–143.7)	140.0 (136.1–143.8)	140.1 (136.3–143.7)	0.744
Chloride (mmol/L)	103.7 (99.4–107.9)	102.7 (98.9–107.5)	104.6 (100.2–108.4)	0.010
Calcium (mmol/L)	2.01 (1.89–2.13)	2.00 (1.88–2.13)	2.02 (1.90–2.13)	0.500
Blood urea nitrogen (mmol/L)	7.79 (5.14–12.91)	7.51 (4.90–12.30)	8.57 (5.52–13.82)	0.074
Creatinine (μmol/L)	86.0 (58.8–145.3)	87.0 (60.3–147.8)	85.0 (57.0–143.0)	0.593
Estimated glomerular filtration rate (ml/min)	79.9 (41.3–108.4)	77.9 (38.5–107.7)	83.2 (44.1–109.5)	0.465

Quantitative variables are expressed as medians (interquartile ranges). Categorical variables were reported as number of events (proportions). Abbreviations: ICU, intensive care unit; IQR, interquartile range.

### Primary Outcome

A total of 115 patients in the bicarbonate Ringer’s group and 180 patients in the saline group had a major adverse kidney event within 30 days [42.6 vs. 45.9%, odds ratio (OR) = 0.874; 95% confidence interval (CI), 0.639 to 1.194; *p* = 0.398, adjusted OR = 0.907, 95% CI, 0.626 to 1.315; *p* = 0.607]. Compared to the saline group, the bicarbonate Ringer’s group had a significant lower rate of the new receipt of renal replacement therapy in MAKE30 (35.7 vs. 25.6%, OR = 0.618; 95% CI, 0.439 to 0.871; *p* = 0.006; adjusted OR = 0.651, 95% CI, 0.436 to 0.972; *p* = 0.036), especially among survivors (31.5 vs. 18.2%, OR = 0.483; 95% CI, 0.317 to 0.737; *p* = 0.001; adjusted OR = 0.539, 95% CI, 0.334 to 0.869; *p* = 0.011). There was no significant difference between the two groups in 30-day mortality and final creatinine level ≥200% of baseline (Table 2). The results of binary logistic regression revealed a significant linearity relationship between outcomes and covariates, while the results of collinearity diagnostic analysis revealed no multi-collinearity between these covariates in the multivariates logistic regression models (Supplementary Tables S1, S2). The detailed fluid administration regimens, including purpose, amount, timing and duration were showed in Supplementary Figure S1.

**TABLE 2:** Primary and secondary outcomes.

Outcome	Total (n = 662)	Saline (n = 392)	Bicarbonate Ringer’s (n = 270)	Odds ratio (95% CI)	*p*-value	Adjusted odds ratio (95%CI)^a^	*p*-value
Primary outcome							
Major adverse kidney event within 30 days, no. (%)	295 (44.6)	180 (45.9)	115 (42.6)	0.874 (0.639–1.194)	0.398	0.907 (0.626–1.315)	0.607
Components of primary outcome							
30-day mortality, no. (%)	126 (19.0)	65 (16.6)	61 (22.6)	1.468 (0.994–2.169)	0.054	1.428 (0.929–2.196)	0.104
New receipt of renal replacement therapy, no. (%)	209 (31.6)	140 (35.7)	69 (25.6)	0.618 (0.439–0.871)	0.006	0.651 (0.436–0.972)	0.036
Among survivors, no. (%)	141/536 (26.3)	103/327 (31.5)	38/209 (18.2)	0.483 (0.317–0.737)	0.001	0.539 (0.334–0.869)	0.011
Final creatinine level ≥200% of baseline, no. (%)	142 (21.5)	87 (22.2)	55 (20.4)	0.897 (0.613–1.312)	0.574	0.876 (0.557–1.376)	0.565
Among survivors, no. (%)	95/536 (17.7)	61/327 (18.7)	34/209 (16.3)	0.847 (0.534–1.343)	0.481	1.063 (0.618–1.829)	0.825
Among survivors without new renal replacement therapy, no. (%)	28/395 (7.1)	12/224 (5.4)	16/171 (9.4)	1.824 (0.839–3.965)	0.129	1.916 (0.806–4.559)	0.141
Secondary outcomes							
Hospital stay (days)	17.0 (7.0–28.0)	17.0 (8.0–28.0)	15.5 (7.0–29.3)	—	0.654	—	—
Length of ICU stay (days)	5.0 (3.0–9.0)	4.0 (3.0–8.0)	5.0 (3.0–10.0)	—	0.045	—	—
Vasopressor days	1.0 (0–2.0)	1.0 (0–2.0)	1.0 (0–2.0)	—	0.506	—	—
New renal replacement therapy days	0 (0–2.0)	0 (0–2.0)	0 (0–1.0)	—	0.012	—	—
Among new renal replacement therapy (days)	3.0 (2.0–4.0)	2.5 (2.0–4.0)	3.0 (2.0–4.0)	—	0.340	—	—
Mechanical ventilation days	1.0 (0–4.0)	1.0 (0–4.0)	1.0 (0–4.0)	—	0.469	—	—
ICU-free days	9.0 (0–19.3)	10.0 (0–20.8)	7.0 (0–18.0)	—	0.414	—	—
Ventilator-free days	14.0 (5.0–25.0)	14.5 (5.0–25.0)	13.0 (5.0–25.0)	—	0.792	—	—
Vasopressor-free days	15.0 (6.0–27.0)	16.0 (6.0–26.0)	13.5 (5.0–28.0)	—	0.741	—	—
New renal replacement therapy-free days	15.0 (7.0–28.0)	16.0 (6.3–27.0)	15.0 (7.0–28.0)	—	0.989	—	—
Among new renal replacement therapy (days)	11.0 (2.0–21.0)	11.0 (3.0–20.0)	8.0 (1.0–27.0)	—	0.895	—	—
Cumulative fluid volume for resuscitation or maintenance within 24 h (ml)	500 (500–1,000)	500 (500–1,000)	500 (500–1,000)	—	0.902	—	—

a Adjusted by age, gender, admission source, SOFA score at ICU admission and diagnosis on ICU admission, including medical diseases, cardiovascular disease, neurological disease, traumatic, surgical, sepsis, and traumatic brain injury. Quantitative variables are expressed as medians (interquartile ranges). Categorical variables were reported as number of events (proportions). Abbreviations: ICU, intensive care unit; SOFA, sequential organ failure assessment.

In the subgroup of patients with septic shock, similar results were found. In this subgroup, compared to the patients in the saline group, the patients in the bicarbonate Ringer’s group had a significant lower rate of the new receipt of renal replacement therapy (67.8 vs. 42.0%, OR = 0.344; 95% CI, 0.167 to 0.709; *p* = 0.004; adjusted OR = 0.260, 95% CI, 0.097 to 0.697; *p* = 0.007), especially among survivors (60.7 vs. 23.3%, OR = 0.197; 95% CI, 0.073 to 0.531; *p* = 0.001; adjusted OR = 0.233, 95% CI, 0.065 to 0.831; *p* = 0.025) (Supplementary Table S3).

**FIGURE 2:**
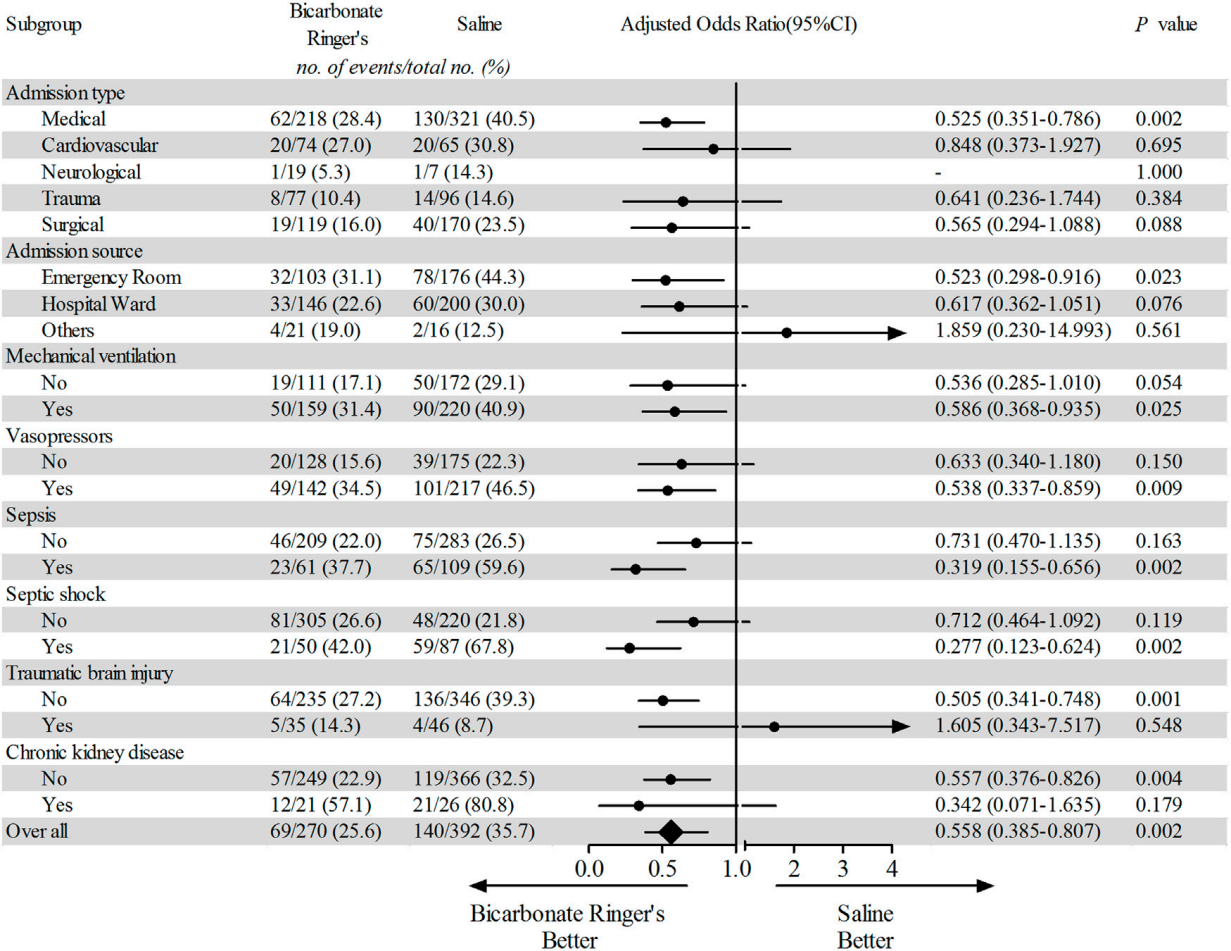
Subgroup analysis of incidence for new receipt of renal replacement therapy. Forest plot shows the number, proportion, adjusted odds ratio and 95% confidence interval of all and subgroups’ patients receiving new receipt of renal replacement therapy in the bicarbonate Ringer’s group and the saline group. The categorical outcomes were analyzed using the logistic regression model by adjusting the age, gender and sequential organ failure assessment (SOFA) score.

In other subgroup analyses, we found that patients with medical admission type, admission from an emergency room, with mechanical ventilation, with vasopressors application, with sepsis, with traumatic brain injury and with chronic kidney disease might benefit from bicarbonate Ringer’s solution in the outcome of new receipt of renal replacement therapy (Figure 2). It was worth noting that the incidence of MAKE30 was higher in the bicarbonate Ringer’s group than that of the saline group among the patients with cardiovascular disease (Supplementary Figures S2–S4).

### Secondary Outcome

The median length of ICU stay of the study population was 5.0 days (IQR, 3.0–9.0). The length of ICU stay in the bicarbonate Ringer’s group was longer than that of the saline group (median, 5.0; IQR, 3.0 to 10.0 vs. median, 4.0; IQR, 3.0 to 8.0; *p* = 0.045) but the new renal replacement therapy days in the bicarbonate Ringer’s group was shorter than that of the saline group (median, 0; IQR, 0 to 1.0 vs. median, 0; IQR, 0 to 2.0; *p* = 0.012) (Table 2). In the subgroup of patients with septic shock, the patients in the bicarbonate Ringer’s group had a shorter duration of vasopressor days and new renal replacement therapy days (Supplementary Table S3).

### Safety Endpoints

**FIGURE 3:**
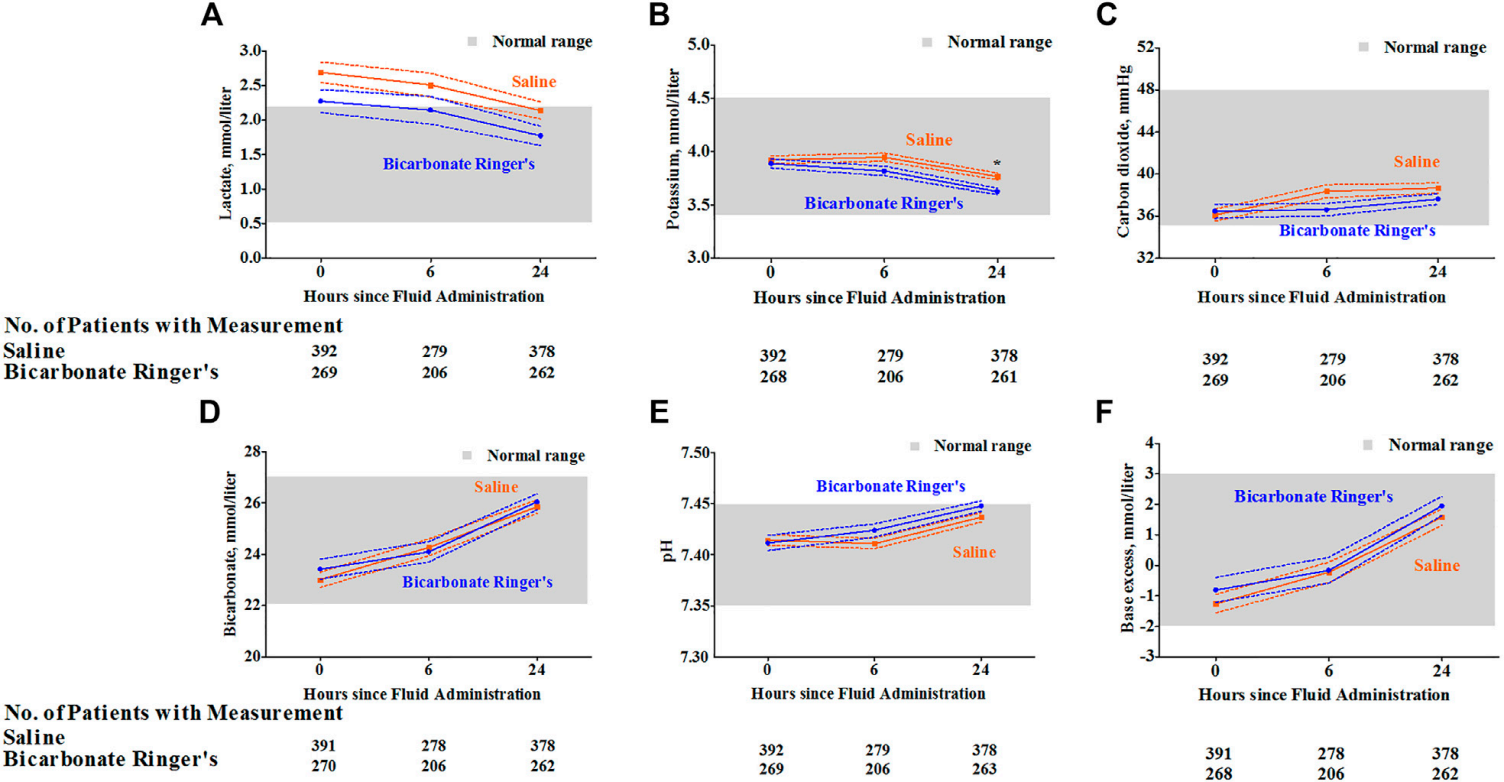
Change of arterial blood gas analysis of each group after the fluid administration. The mean and standard error of arterial blood gas analysis including lactate **(A)**, potassium **(B)**, partial pressure of carbon dioxide **(C)**, plasma concentration of bicarbonate **(D)**, pH level **(E)** and base excess **(F)** after the fluid administration were shown for patients in each group. The partial pressure of carbon dioxide [F _time_ (2, 1780) = 5.02; *p* = 0.0067], pH level [F _time_ (2, 1781) = 14.88; *p* < 0.0001], base excess [F _time_ (2, 1777) = 35.25; *p <* 0.0001], plasma concentration of bicarbonate [F _time_ (2, 1779) = 34.61; *p <* 0.0001], potassium [F _time_ (2, 1778) = 18.05; *p <* 0.0001] and lactate [F _time_ (2, 1778) = 5.77; *p* = 0.0032] were changed over time. Differences between bicarbonate Ringer’s group and saline group were only observed on lactate [F _fluid type_ (1, 1780) = 8.75; *p* = 0.0031] and potassium [F _fluid type_ (1, 1778) = 10.57; *p* = 0.0012]. Bonferroni’s post hoc test was performed for each time point, respectively, and the significance was highlighted by asterisk (* stands for *p <* 0.05).

Over 24 h after the administration of either sodium bicarbonate Ringer’s solution or saline for each group, the plasma lactate levels were decreased to the normal range for both groups (Figure 3A). The plasma lactate levels of Ringer’s group were significantly lower than that of the saline group [F _fluid type_ (1, 1780) = 8.75; *p* = 0.0031]. Arterial blood gas analysis parameters, including partial pressure of carbon dioxide, pH level, base excess, and plasma concentration of bicarbonate were similar between the two groups and were all within the normal range (Figure 3 and Supplementary Tables S4–S7). The plasma concentration of potassium of the saline group was significantly higher than that of bicarbonate Ringer’s group [F _fluid type_ (1, 1778) = 10.57; *p* = 0.0012], but the mean values and standard error ranges of both groups were within the normal range (Figure 3B

**FIGURE 4:**
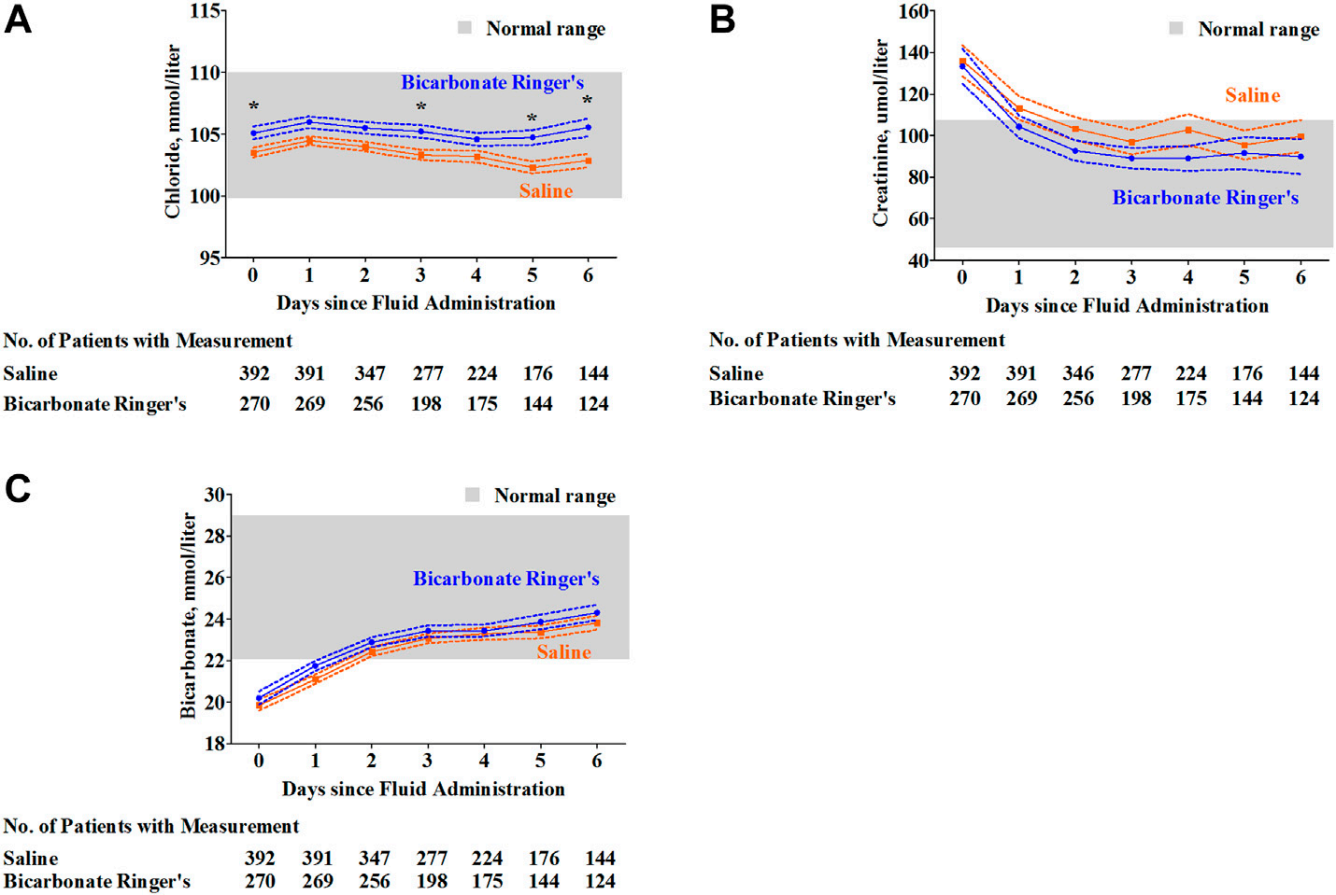
Plasma chloride, creatinine and bicarbonate concentration of each group over seven days after the fluid administration. Over 6 days after the administration of either fluid for each group, the mean and standard error for the measurement of blood biochemical indexes including chloride **(A)**, creatinine **(B)** and bicarbonate **(C)**. Although the mean value of plasma chloride levels was within the normal range, a significant difference in plasma chloride levels was observed between the saline group and bicarbonate Ringer’s group [F _fluid type_ (3, 3373) = 48.00; *p* < 0.0001], post hoc Bonferroni test revealed significant difference on 0th day (*p* < 0.05), 3rd day (*p* < 0.05), 5th day (*p* < 0.05) and 6th day (*p* < 0.05), respectively **(A)**. Plasma creatinine levels were significantly decreased from an abnormal range to a normal range for both groups [F _time_ (6, 3372) = 8.46; *p* < 0.0001] from 0th day to 6th day. A significant difference in plasma creatinine levels was observed between the saline group and bicarbonate Ringer’s group [F _fluid type_ (1, 3372) = 4.44; *p* = 0.0352], but the post hoc Bonferroni test revealed no significant differences between groups at any time point **(B)**. Plasma bicarbonate levels were increased from relative acidic level (lower than 22.0 mmol/L) to the normal range from 0th day to 6th day [F _time_ (6, 3373) = 46.78; *p* < 0.0001]. A significant difference in plasma bicarbonate levels was observed between the saline group and bicarbonate Ringer’s group [F _fluid type_ (1, 3373) = 6.96; *p* = 0.0084], but the post hoc Bonferroni test revealed no significant differences between groups at any time point **(C)**.

Over 6 days after the administration of either fluid for each group, despite significant differences in plasma creatinine and bicarbonate concentration between groups over time, the plasma creatinine and bicarbonate concentration were optimized to the normal range over time for both groups (Figures 4B,C). Post hoc tests revealed no significant differences at any time point. Although results showed that there was a significant difference in plasma chloride concentration between groups [F _fluid type_ (1, 3373) = 48.00; *p* < 0.0001], the mean values and standard error ranges of both groups were within the normal range (Figure 4A). In the subgroup of patients with septic shock, similar results were found in these repeated measurement data of arterial blood gas analysis and blood biochemical indexes (Supplementary Figures S5, S6).

Furthermore, to determine the safety outcome, detailed subgroup analyses were carried out. For patients in the bicarbonate Ringer’s group, we did not observe any significant increase of partial pressure of carbon dioxide in patients with chronic obstructive pulmonary disease, patients with or without mechanical ventilation over the 24 h after the fluid administration (Supplementary Table S4). Similarly, no significant increase of bicarbonate concentration, base excess concentration or pH level was observed for patients in the bicarbonate Ringer’s group in the subgroups, including the patients with chronic kidney disease, with or without new renal replacement therapy (Supplementary Tables S5–S7).

## Discussion

Several studies have shown that the balance solution may be more beneficial to patients (Young et al., 2015; Semler et al., 2017; Barea-Mendoza et al., 2018). There are many kinds of balanced crystalloids. At present, the most widely used is sodium lactate Ringer’s solution, sodium acetate Ringer’s solution and sodium bicarbonate Ringer’s solution. Sodium lactate Ringer’s solution contains lactic acid, which may interfere with the diagnosis and judgment of the disease (Hadimioglu et al., 2008). Lactic acid is mainly oxidized in the liver and kidney and then metabolized through the tricarboxylic acid cycle (Rohrig et al., 2014). Thus, sodium lactate Ringer’s solution may increase the burden of the liver and kidney function (Shin et al., 2011). The acetate in sodium acetate Ringer’s solution can be metabolized by the tricarboxylic acid cycle in a variety of organ and tissue cells (Gille et al., 2014; Pfortmueller and Fleischmann, 2016). In a case report of resuscitation with sodium acetate, it was found that acetate metabolism decreased and lactate increased in patients with low blood volume and liver hypoperfusion (McCague et al., 2012). Acetic acid has a vasodilator effect, and an inhibitory effect on the cardiovascular system if it is infused rapidly and heavily (Jacob et al., 1997). Therefore, sodium acetate Ringer’s solution may also cause adverse clinical consequences in certain patients. Sodium bicarbonate Ringer’s solution, as a relatively newly emerging balance solution, has been widely used in clinical practice in recent years. Some scholars proposed that sodium bicarbonate Ringer’s solution has good application prospects in fluid resuscitation of sepsis, perioperative period, hemorrhagic shock and other clinical use (Shimada et al., 2005; Oikawa et al., 2015; Hongo et al., 2021; Ma et al., 2021). But its safety and efficacy need further research, especially among critically ill patients in the real world. At present, there are few clinical studies on the efficacy and safety of sodium bicarbonate Ringer’s solution in the field of critical illness. We conducted this retrospective cohort study on the prognosis and safety of sodium bicarbonate Ringer’s solution in critically ill patients.

A recently published study showed that balanced crystalloids (sodium lactated Ringer’s solution or sodium acetate Ringer’s solution) could reduce the incidence of major adverse kidney event within 30 days compared with saline (Barea-Mendoza et al., 2018). In their study, the major adverse kidney event with 30 days was significantly lower in the balanced crystalloids group than that of the saline group among critically ill adults (Barea-Mendoza et al., 2018). Until now, rare studies were carried out among the critically ill adults or shock patients to compare the patients’ outcomes and renal function between the sodium bicarbonate Ringer’s solution and saline. In our study, similar results were found. Despite no statistically significant difference in general major adverse kidney events was found between the sodium bicarbonate Ringer’s solution group and saline group, the incidence of receiving new renal replacement therapy in bicarbonate Ringer’s group was significantly lower than that of the saline group (Table 2).

However, in the subgroup analysis by stratifying patients with admission type, admission source, different diagnosis and major treatments, we found that the patients in the bicarbonate Ringer’s group had a higher incidence of the major adverse event among patients with cardiovascular admission type (Supplementary Figure S2). This also reminds us the need to pay attention to the patients with cardiovascular admission type when using sodium bicarbonate Ringer’s solution.

As bicarbonate can directly dissociate from sodium bicarbonate without metabolic process, it is thought that contained sodium bicarbonate in the sodium bicarbonate Ringer’s solution is the most biologically suitable alkalinizing reagent for alkalization effect during shock or acidosis status in the clinic. The primary studies based on the shock animal models have shown that sodium bicarbonate Ringer’s solution significantly improved blood base excess values faster and more markedly than did sodium lactate Ringer’s solution and Ringer’s solution (Satoh et al., 2005a). Similar results were validated in the perioperative solution study of rabbits with partially hepatectomized rabbits from the same research team (Satoh et al., 2005b). In our study, pH level, base excess and bicarbonate concentration were optimized within 24 h after the infusion of sodium bicarbonate Ringer’s solution or saline, respectively. However, the superiority in alkalization of sodium bicarbonate Ringer’s solution compared to the saline was not observed in our study among these critically ill patients. In a small-size randomized controlled clinical trial among abdominal aortic aneurysm repair patients, pH level and base excess were also similar between sodium bicarbonate Ringer’s solution users and sodium acetate Ringer’s solution users during and after the aortic cross-clamping (Shimada et al., 2005). Considering the biochemical and pharmacokinetic characteristics of sodium bicarbonate Ringer’s solution itself, in addition to its favorable alkalinizing effect among patients with acidosis, patients who received a certain amount of this fluid might also suffer a series of adverse events, including elevation of pH level, increasing of bicarbonate and base excess, accumulation of carbon dioxide (Satoh et al., 2005a; Satoh et al., 2005b; Shimada et al., 2005; Oikawa et al., 2015; Hongo et al., 2021; Ma et al., 2021; Wang et al., 2021). Therefore, unexpected results including excessive alkalization should be investigated, especially in critically ill patients with multiple organ dysfunctions, including respiratory failure, acute renal injury or dysfunction. In general, compared with the infusion of sodium acetate Ringer’s solution, sodium lactate Ringer’s solution or Ringer’s solution, the animal model or some patients who infused sodium bicarbonate Ringer’s solution has a normal range of pH levels, base excess, bicarbonate concentration and partial pressure of carbon dioxide (Satoh et al., 2005a; Satoh et al., 2005b; Wang et al., 2021). To determine the safety of this fluid among critically ill patients, we also explored whether acid–base imbalance occurred in our study. We found that the use of sodium bicarbonate Ringer’s solution did not cause acid–base balance disturbances and accumulation of carbon dioxide, even in high-risk patients, including patients with chronic kidney disease or chronic obstructive pulmonary disease, patients with or without renal replacement therapy, and patients with mechanical ventilation (Supplementary Tables S4–S7). These acid–base balance-related variables, as well as plasma concentration of creatinine and chloride, were monitored for 24 h and some for 7 days in our study. In general, we found that these safety-related variables were stable and kept within the normal ranges for both groups (Figures 3, 4) even in the subgroup of patients with septic shock (Supplementary Figures S5, S6).

Our study has several limitations. The first limitation is the nature of a retrospective single-centered study and a relatively small sample size. Although we excluded patients who received both saline and bicarbonate Ringer’s solution for fluid resuscitation or maintenance from the analysis, patients in the bicarbonate Ringer’s group still received a small amount of saline for medication injection. As a result, randomized controlled trials are pending in the future by comparing patients who only use bicarbonate Ringer’s solution with patients who only use saline. Second, in the multivariate logistic regression analysis, we conducted linearity analysis between outcome and covariates and collinearity diagnostic analysis between covariates. However, a newly developed ensemble modeling that can address non-linearity automatically without pre-specification is a potential advanced approach to resolving this problem (Zhang et al., 2022). It is valuable to apply this modeling in further studies. Third, the use of fluid is a time-varying exposure that can cause bias in the current results. The marginal cox model, time-dependent propensity score methods and other time-varying models might help in resolving this problem (Zhang et al., 2018; Zhang et al., 2020; Ripa et al., 2021). However, these models only account for time-varying exposure in the exposure group since the study design of these studies actually had no exposure in the control group. In our study, the saline group received a similar amount of saline for resuscitation or maintenance also in a time-varying manner, which cannot be accounted as “blank control.” Further advanced model, which can handle one time-varying exposure in the exposure group and another time-varying exposure in the control group simultaneously, is pending.

## Conclusion

Compared to patients using saline as fluid resuscitation or maintenance treatment, patients using sodium bicarbonate Ringer’s solution had a lower incidence of receiving new renal replacement therapy while having no significant increase in 30-day mortality, persistent renal dysfunction, and major adverse kidney event within 30 days from ICU admission among critically ill patients. Patients in the bicarbonate Ringer’s group had a longer length of ICU stay than the patients in the saline group, but their new renal replacement therapy days were shorter. No acid–base balance disturbances and accumulation of carbon dioxide were observed during the follow-up period after the fluid administration.

## Data Availability

The raw data supporting the conclusion of this article will be made available by the authors, without undue reservation.
