# Supplementary figures and images for: The Efficacy and Safety of Sodium Bicarbonate Ringer’s Solution in Critically Ill Patients: A Retrospective Cohort Study
Source: Front Pharmacol. 2022 Mar 30;13:829394. doi: 10.3389/fphar.2022.829394 (PMC9006048; doi:10.3389/fphar.2022.829394)

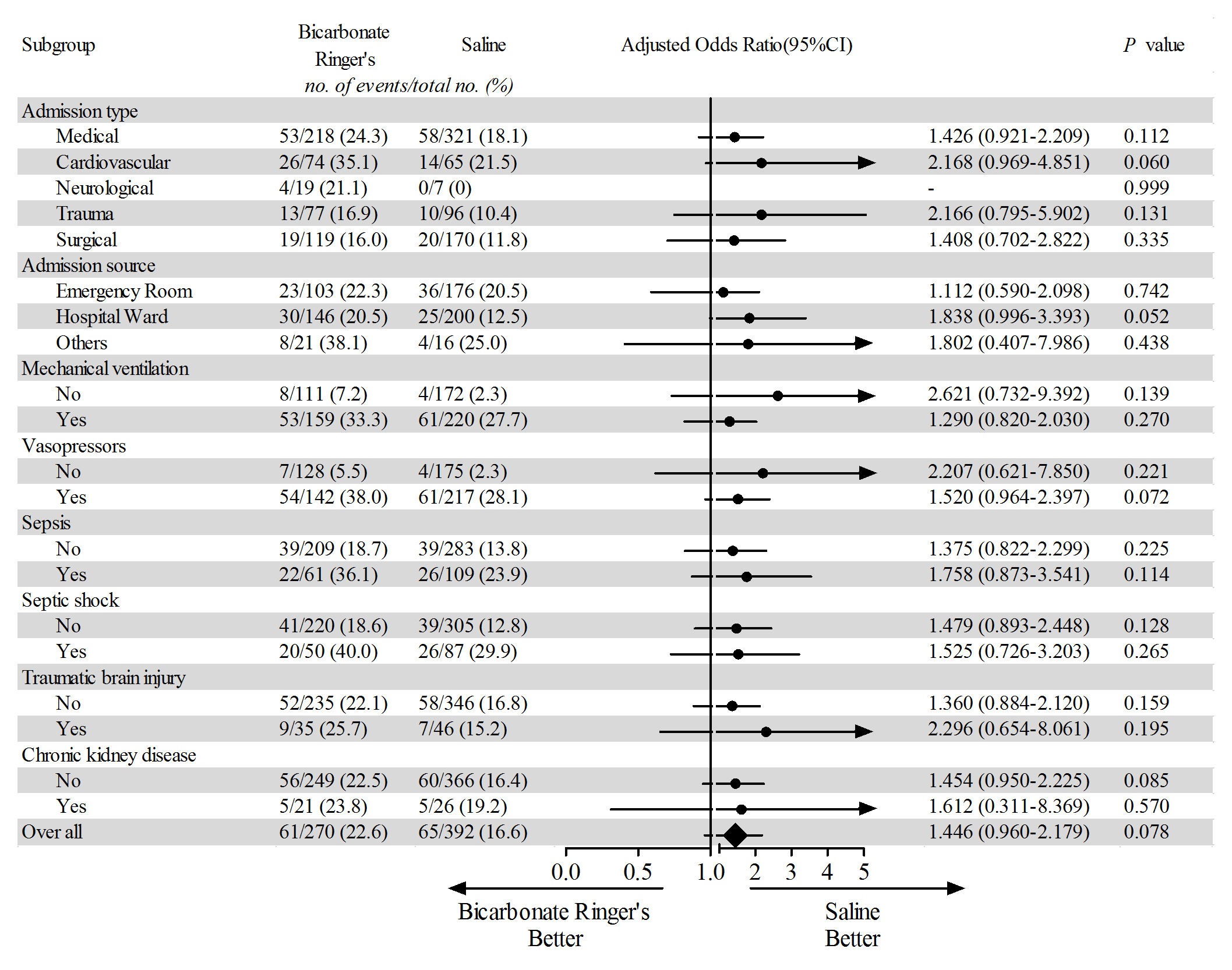

Supplement: Supplementary file 1 [file Image3.jpg]

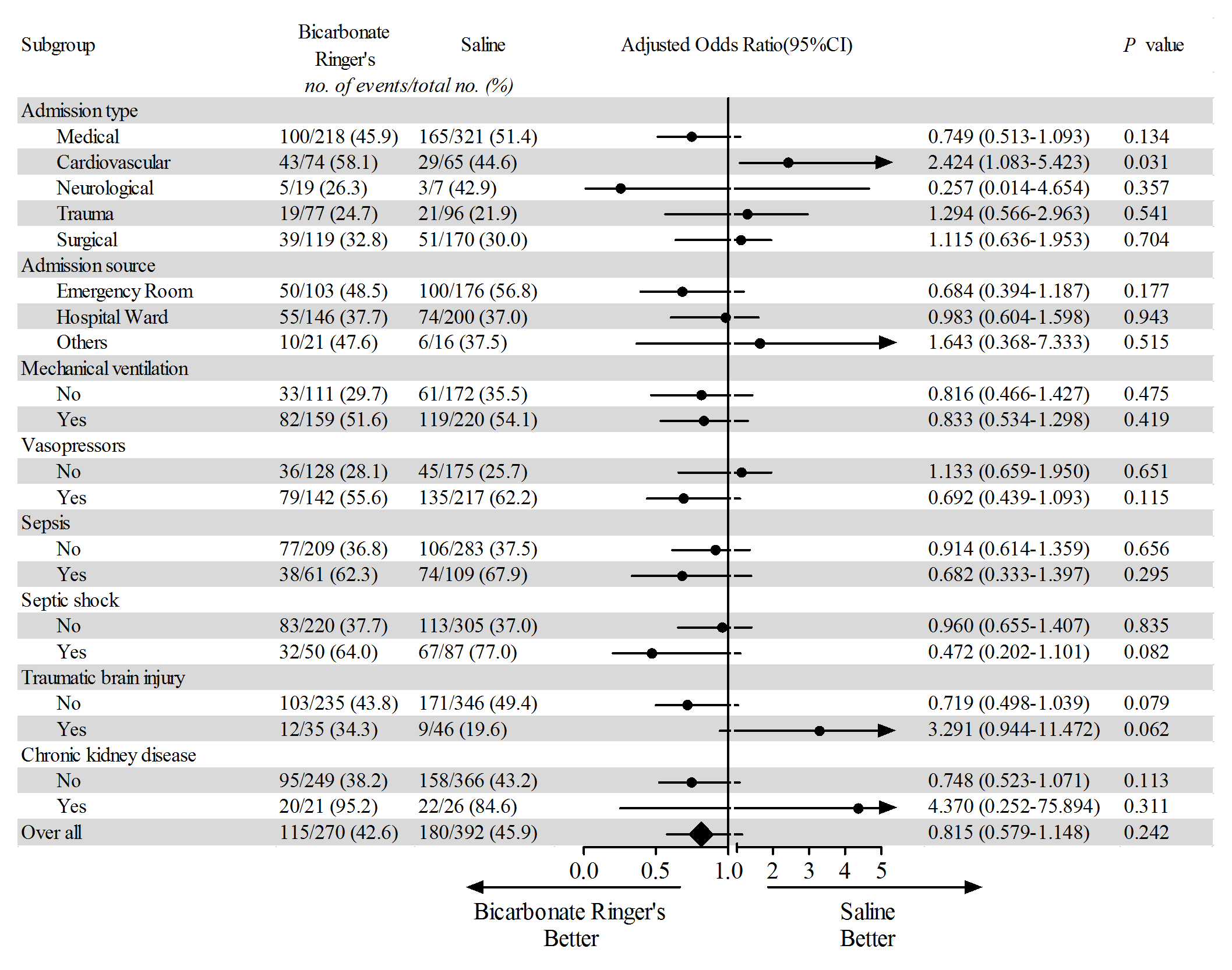

Supplement: Supplementary file 2 [file Image2.jpg]

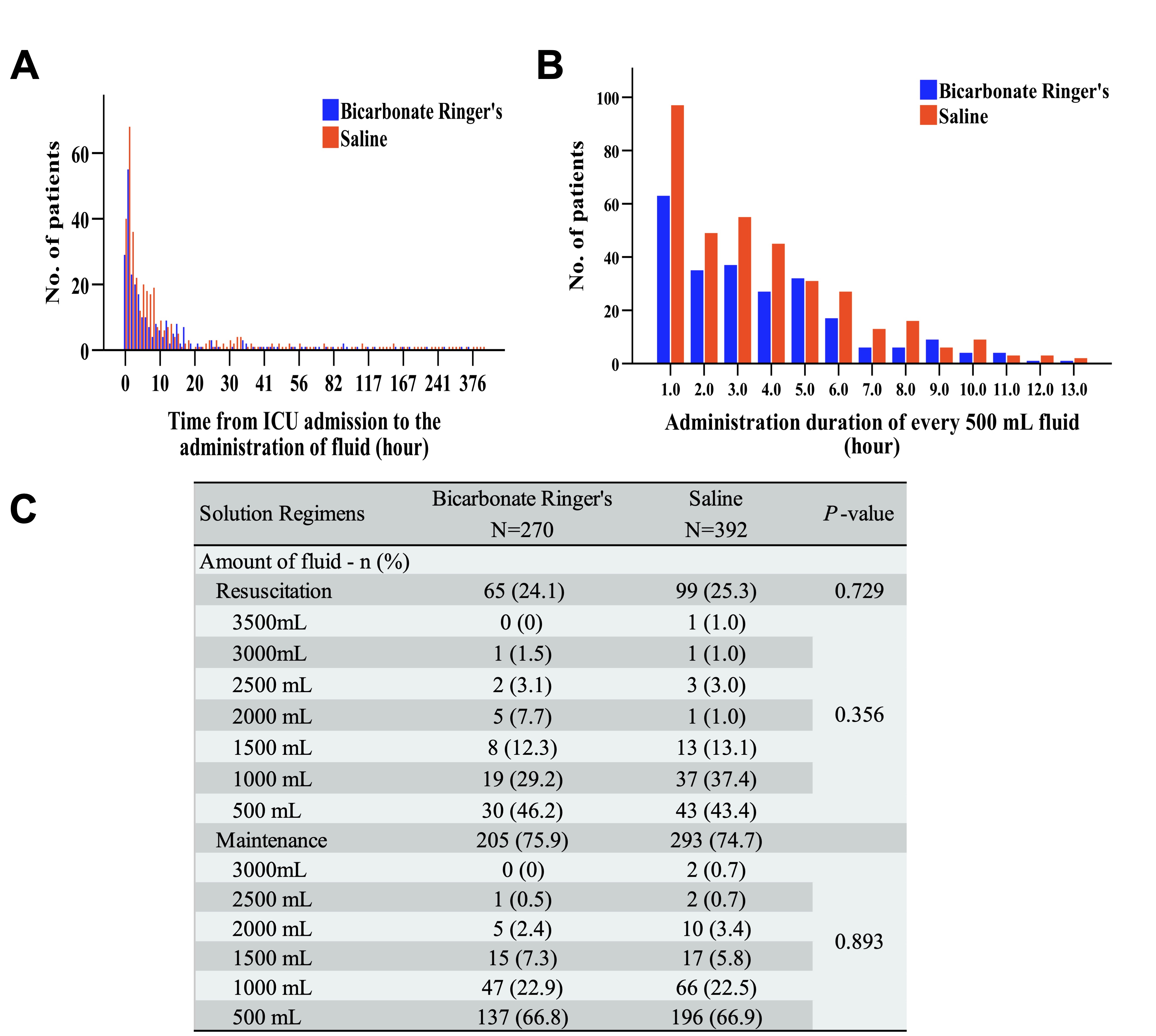

Supplement: Supplementary file 3 [file Image1.JPEG]

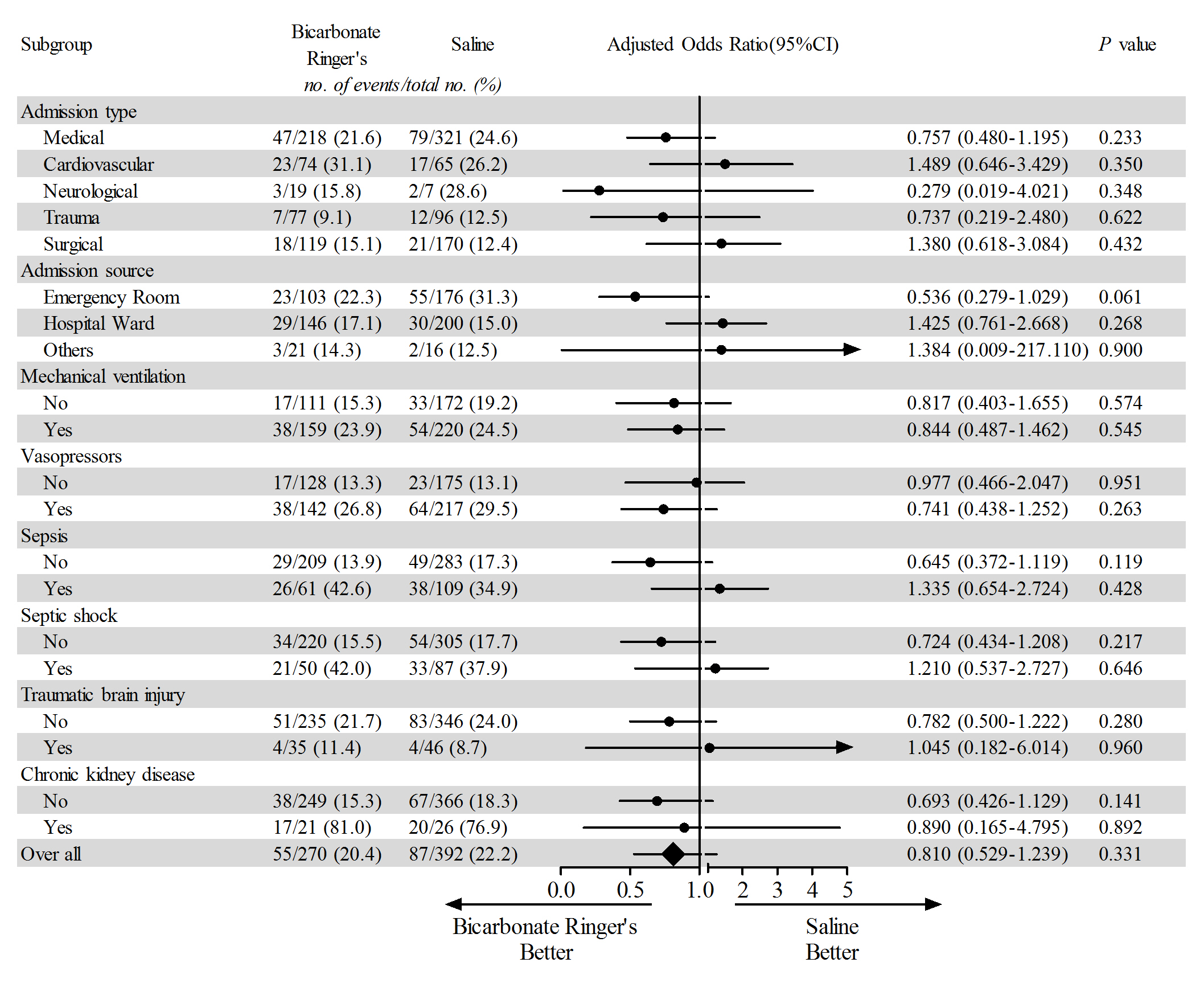

Supplement: Supplementary file 4 [file Image4.JPEG]

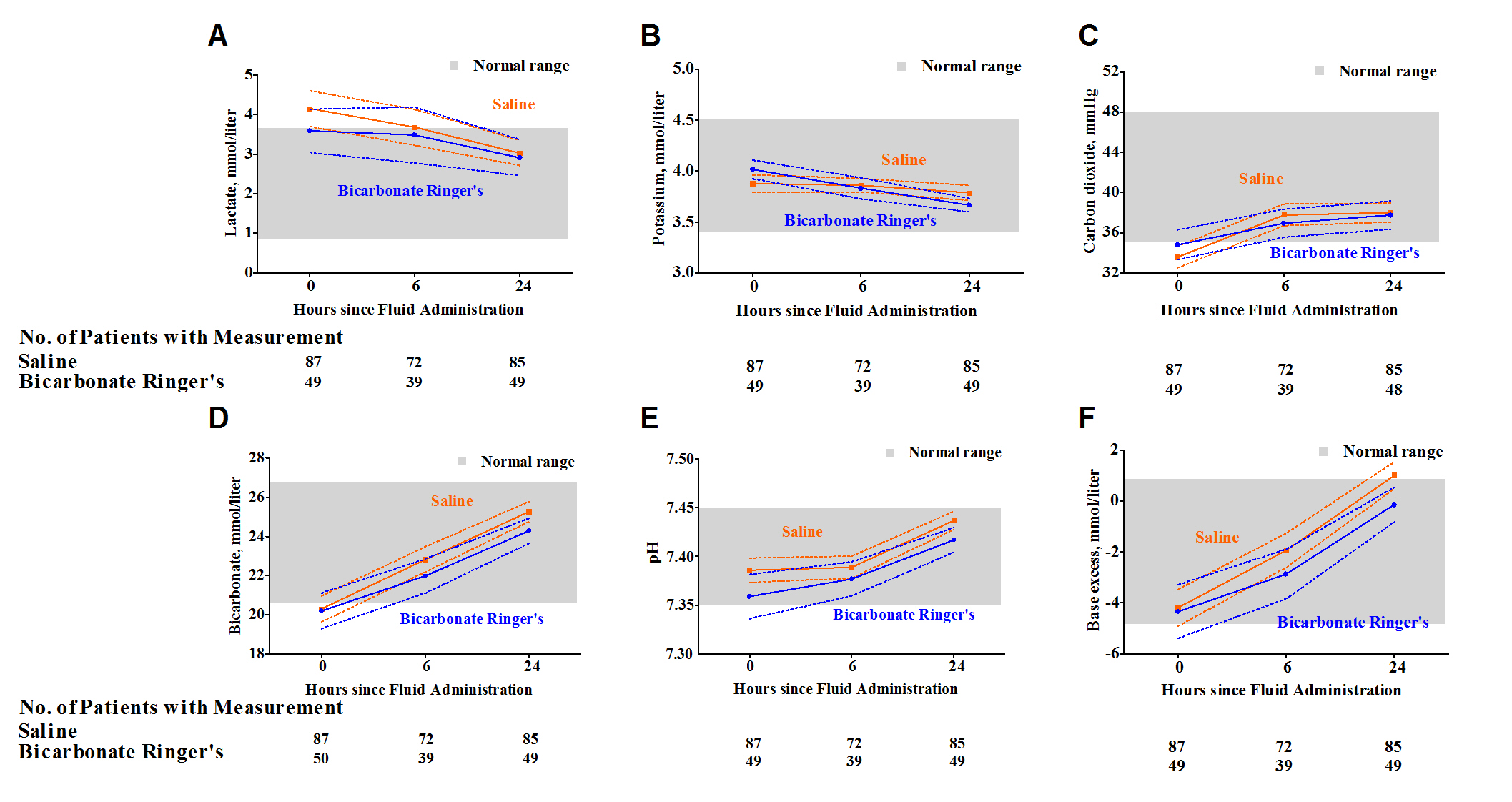

Supplement: Supplementary file 5 [file Image5.JPEG]

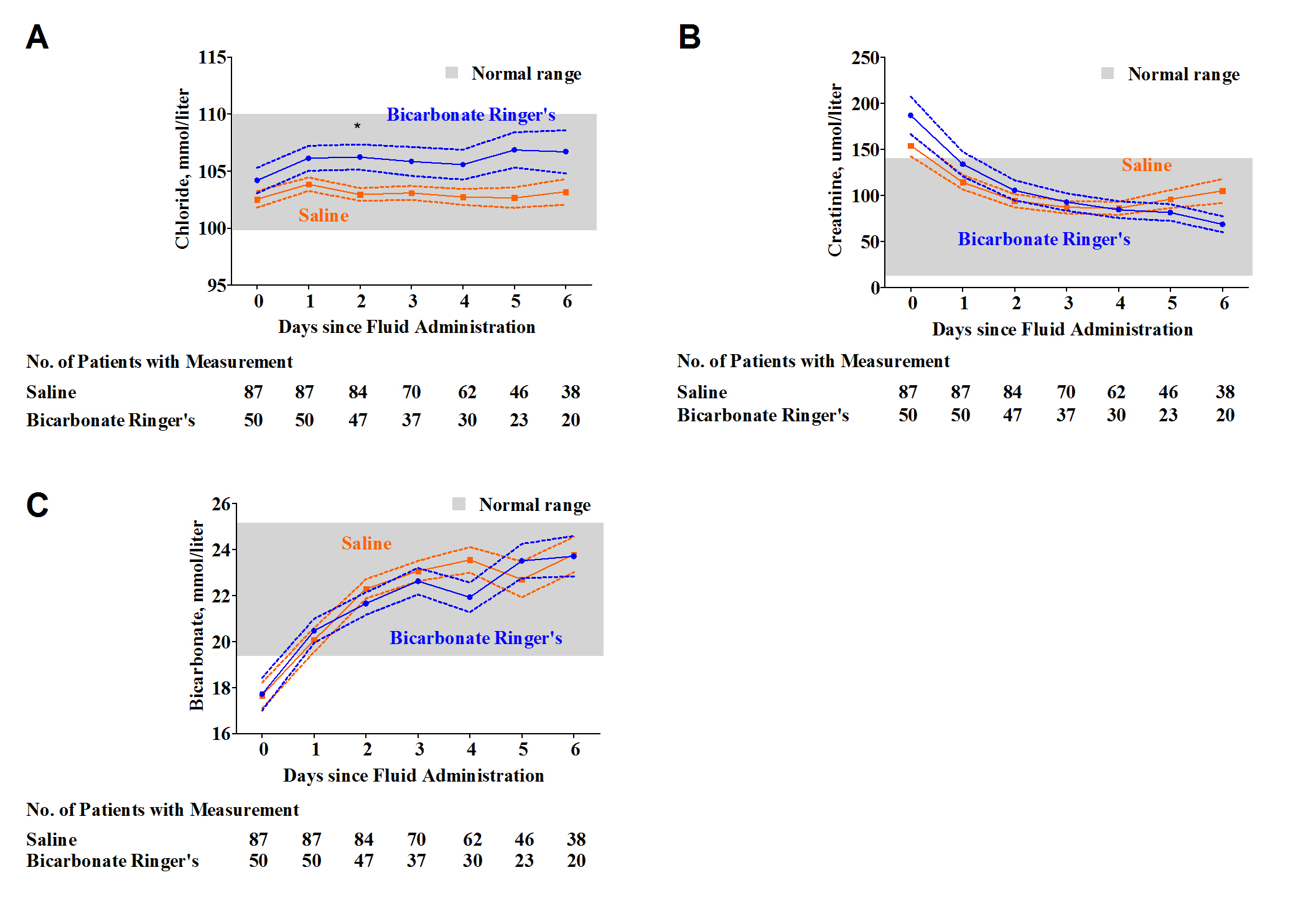

Supplement: Supplementary file 7 [file Image6.JPEG]
