# Supplementary material for: The Efficacy and Safety of Sodium Bicarbonate Ringer’s Solution in Critically Ill Patients: A Retrospective Cohort Study
Source: Front Pharmacol. 2022 Mar 30;13:829394. doi: 10.3389/fphar.2022.829394 (PMC9006048; doi:10.3389/fphar.2022.829394)
Supplement: Supplementary file 6 [file DataSheet1.docx]

**Figure S1. Details of fluids administration.**


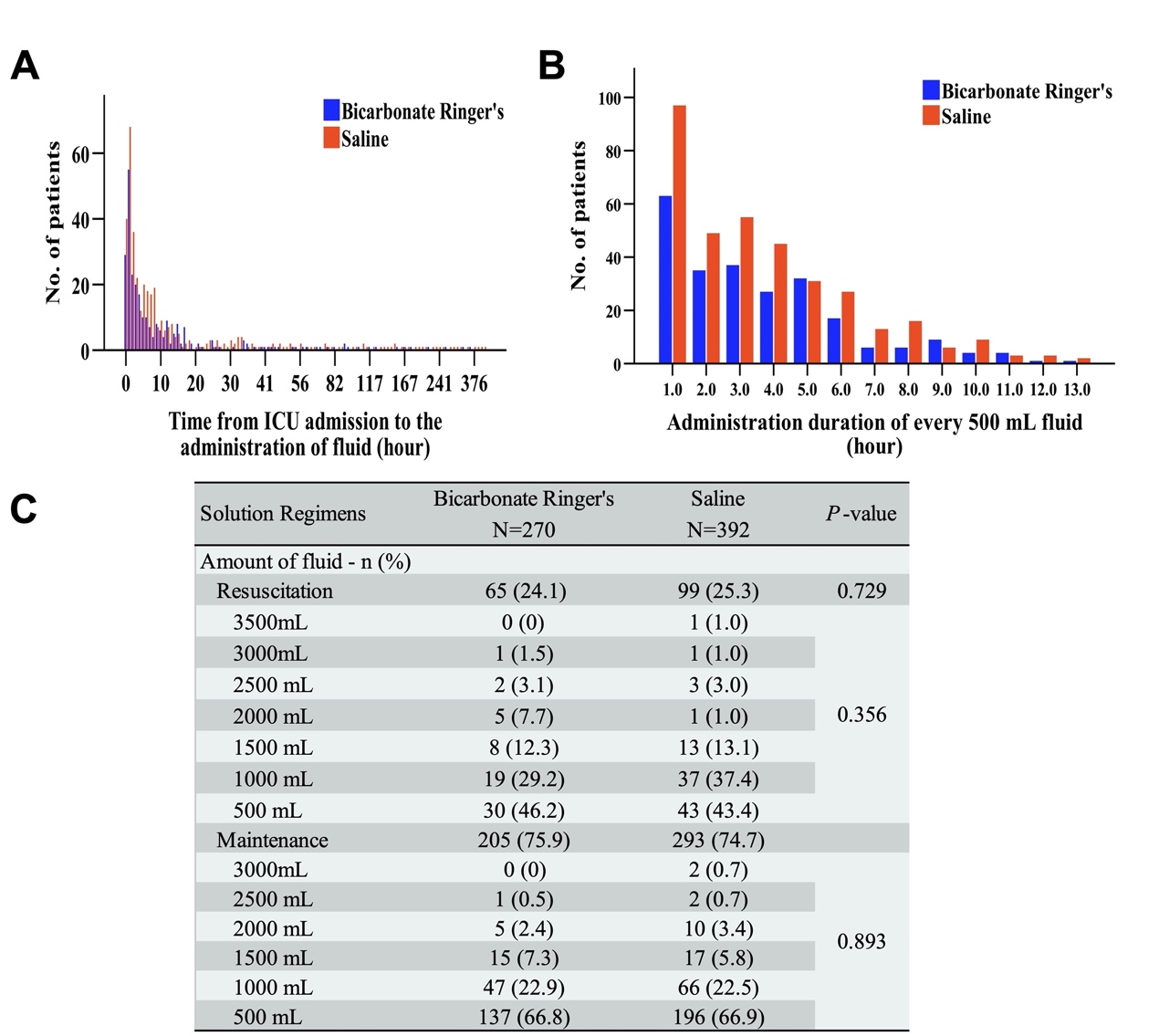


(A) Histogram of time (in hours) from ICU admission to the administration of fluid. The median time in the bicarbonate Ringer’s solution group and the saline group is 4 hours (IQR 1~13) and 5 hours (IQR 1~15) respectively, with no significant difference between the two groups (Mann-Whitney test, *P* = 0.152).

(B) Histogram of administration duration (in hours) of every 500 mL fluid. The median duration in the bicarbonate Ringer’s solution group and the saline group is 3.0 hours (IQR 1.8~5.0) and 3.0 hours (IQR 1.0~5.0) respectively, with no significant difference between the two groups (Mann-Whitney test, *P* = 0.696).

(C) Cumulative fluid volume in resuscitation or maintenance purpose for each group stratified by amount.

**Figure S2. Subgroup Analysis of the incidence of MAKE30 from ICU Admission.**


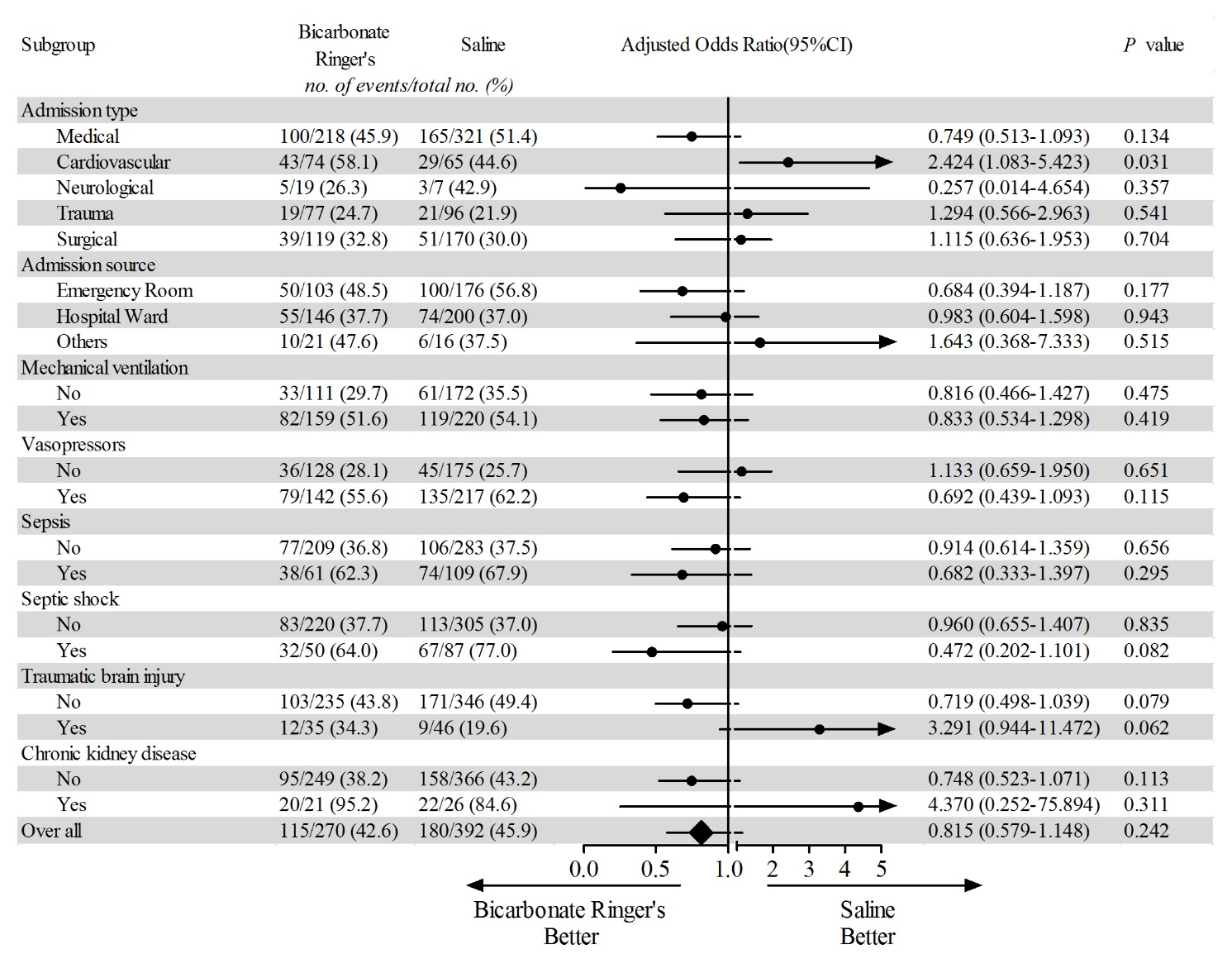


The forest plot showed the number, proportion, adjusted odds ratio and 95% confidence interval of all and subgroups’ patients who met MAKE30 in the bicarbonate Ringer’s group and the saline group. The categorical outcomes were analyzed using the logistic regression model by adjusting the age, gender and sequential organ failure assessment score.

Abbreviation: MAKE30: major adverse kidney event within 30 days, which included death, new receipt of renal-replacement therapy, or persistent renal dysfunction (defined as a final inpatient plasma creatinine value ≥ 200% of the baseline value).

**Figure S3. Subgroup Analysis of the 30-day mortality.**


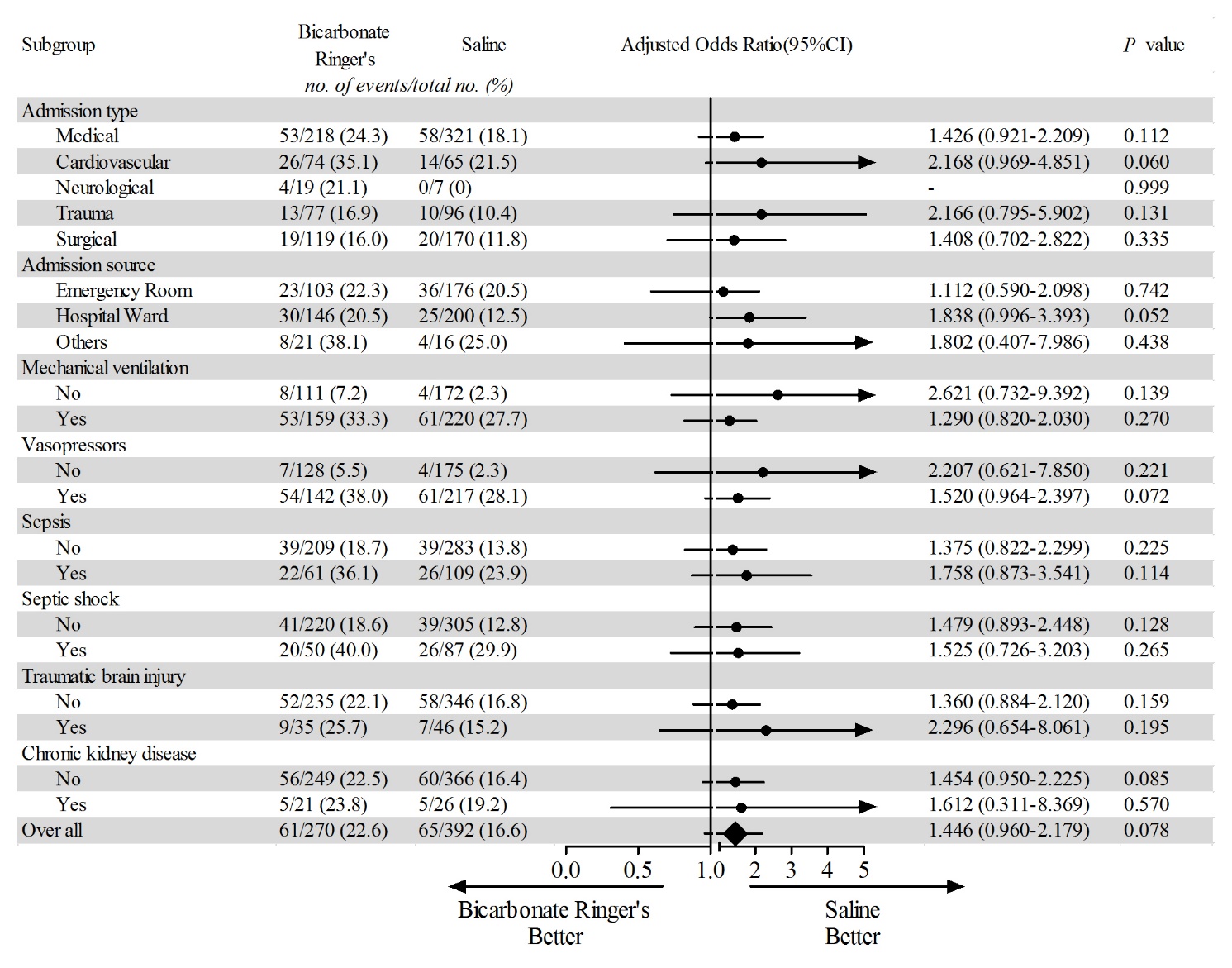


The forest plot showed the number, proportion, adjusted odds ratio and 95% confidence interval of all and subgroups’ patients of the 30-day mortality in the bicarbonate Ringer’s group and the saline group. The categorical outcomes were analyzed using the logistic regression model by adjusting the age, gender and sequential organ failure assessment score.

**Figure S4. Subgroup Analysis of incidence of the Persistent Renal Dysfunction.**


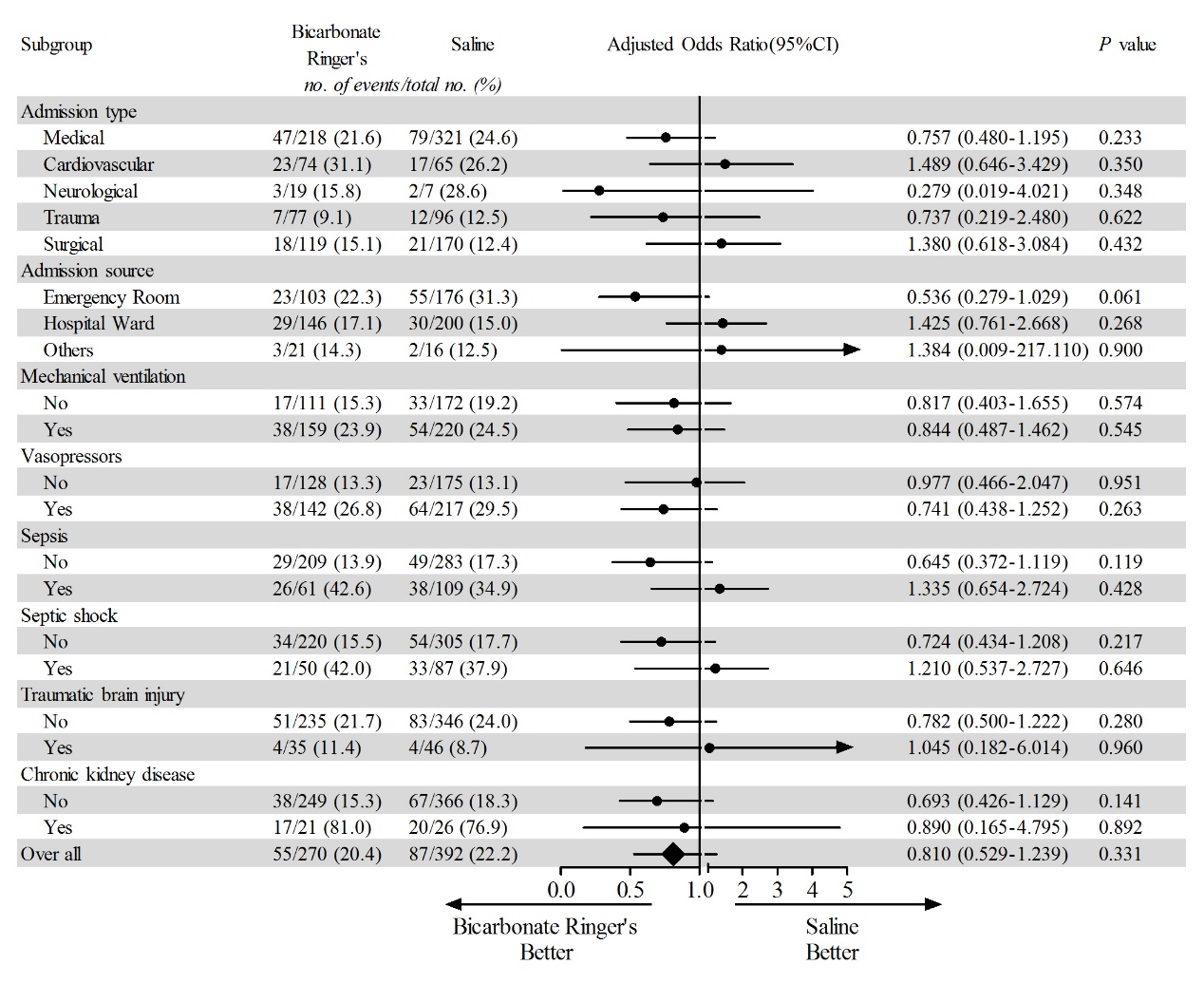


The forest plot showed the number, proportion, adjusted odds ratio and 95% confidence interval of all and subgroups’ patients with persistent renal dysfunction in the bicarbonate Ringer’s group and the saline group. Persistent renal dysfunction was defined as final plasma creatinine level ≥200% of baseline. The categorical outcomes were analyzed using the logistic regression model by adjusting the age, gender and sequential organ failure assessment score.

**Figure S5.** **Change of arterial blood gas analysis after the fluid administration of each group in the subgroup of patients with septic shock.**

**
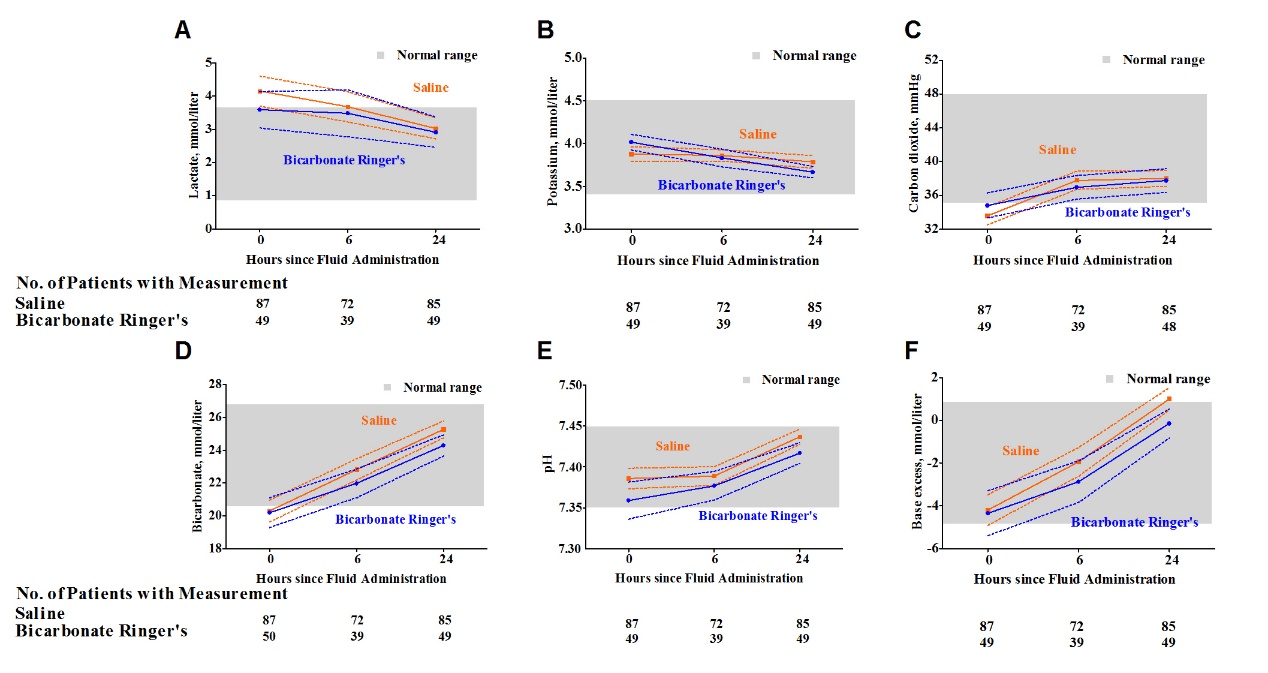
**

In a subgroup of patients with septic shock, the changes of the mean and standard error of arterial blood gas analysis including lactate (Panel A), potassium (Panel B), partial pressure of carbon dioxide (Panel C), the plasma concentration of bicarbonate (Panel D), pH level (Panel E) and base excess (Panel F) after the fluid administration were shown with colored curves. Potassium (F_time_ (2, 375) = 3.34; *P =* 0.0363), partial pressure of carbon dioxide (F_time_ (2, 374) = 5.19; *P* = 0.0060), plasma concentration of bicarbonate (F_time_ (2, 376) = 19.67; *P <* 0.0001), pH level (F_time_ (2, 375) = 8.10; *P* = 0.0004) and base excess (F_time_ (2, 375) = 18.36; *P <* 0.0001) were changed over time. There were no differences between the bicarbonate Ringer’s group and the saline group. Bonferroni post hoc test was performed for each time point respectively and the significance was highlighted by an asterisk.**Figure S6. Plasma chloride, creatinine and bicarbonate concentration of patients in each group among patients with septic shock over seven days after fluid administration.**

**
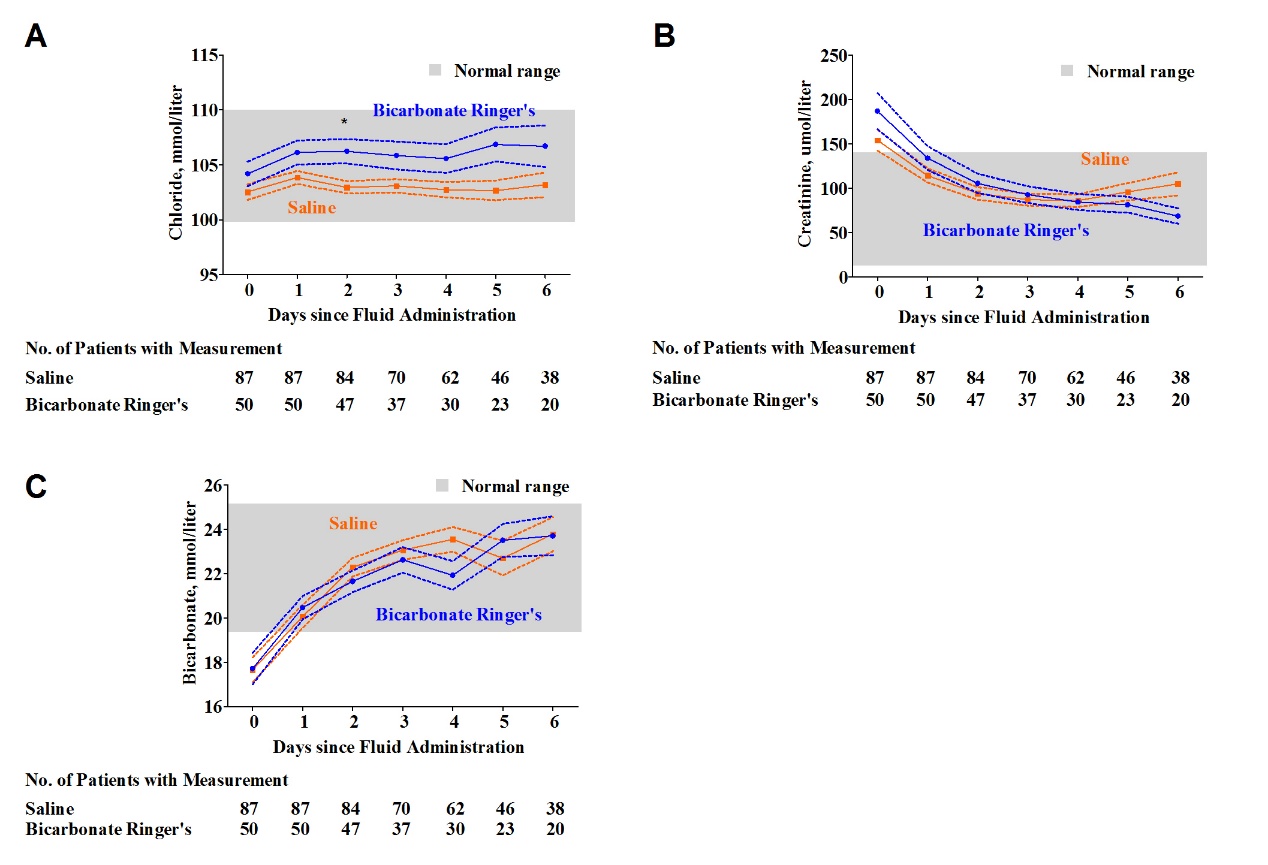
**

Over 6 days after the administration of either fluid for each group, the mean and standard error of blood biochemical indexes including chloride (Panel A), creatinine (Panel B) and bicarbonate (Panel C) were shown. Although the mean value of plasma chloride levels was within the normal range, a significant difference in plasma chloride levels was observed between the saline group and bicarbonate Ringer’s group (F_fluid_ _type_ (1, 717) = 29.95; P < 0.0001), post hoc Bonferroni test revealed significant difference on day 2 (P < 0.05) (Panel A). Plasma creatinine levels were significantly decreased from the abnormal range to a normal range for both groups (F_time_ (6, 717) = 12.83; P < 0.0001) from day 0 to 6th day. There was no significant difference in plasma creatinine levels between the saline group and the bicarbonate Ringer’s group (Panel B). Plasma bicarbonate levels were increased from relative acidic level (lower than 22.0 mmol/liter) to the normal range from day 0 to 6th day (F_time_ (6, 717) = 19.96; P < 0.0001). There was no significant difference in plasma bicarbonate levels between the two groups (Panel C). Bonferroni post hoc test was performed for each time point respectively and the significance was highlighted by an asterisk (* stands for *P <* 0.05).

Table S1. Linearity relationship between categorical outcomes and continuous covariates included in the multivariate logistic regression.

| Outcomes | Covariates | *P-value* |
| --- | --- | --- |
| MAKE30 | Age | 0.948 |
|  | SOFA score | 0.125 |
| 30-day mortality | Age | 0.971 |
|  | SOFA score | 0.775 |
| New receipt of renal-replacement therapy | Age | 0.302 |
|  | SOFA score | 0.027 |
| Among survivors | Age | 0.908 |
|  | SOFA score | 0.073 |
| Final creatinine level ≥200% of baseline | Age | 0.749 |
|  | SOFA score | 0.502 |
| Among survivors | Age | 0.393 |
|  | SOFA score | 0.073 |
| Among survivors without new renal-replacement therapy | Age | 0.516 |
|  | SOFA score | 0.299 |

*Abbreviations: MAKE30, Major adverse kidney event within 30 days; SOFA score, sequential organ failure assessment score.*

Table S2. Diagnostic analysis of multi-collinearity of covariates in the multivariate logistic regression model.

| Covariates | MAKE30 | | 30-day mortality | | New receipt of renal-replacement therapy | | Final creatinine level ≥200% of baseline | |
| --- | --- | --- | --- | --- | --- | --- | --- | --- |
|  | Tolerance | VIF | Tolerance | VIF | Tolerance | VIF | Tolerance | VIF |
| Age | 0.912 | 1.097 | 0.912 | 1.097 | 0.912 | 1.097 | 0.912 | 1.097 |
| Gender | 0.945 | 1.058 | 0.945 | 1.058 | 0.945 | 1.058 | 0.945 | 1.058 |
| Source of admission | 0.761 | 1.315 | 0.761 | 1.315 | 0.761 | 1.315 | 0.761 | 1.315 |
| Medical diseases | 0.677 | 1.477 | 0.677 | 1.477 | 0.677 | 1.477 | 0.677 | 1.477 |
| Cardiovascular disease | 0.918 | 1.089 | 0.918 | 1.089 | 0.918 | 1.089 | 0.918 | 1.089 |
| Neurological disease | 0.958 | 1.044 | 0.958 | 1.044 | 0.958 | 1.044 | 0.958 | 1.044 |
| Traumatic | 0.434 | 2.303 | 0.434 | 2.303 | 0.434 | 2.303 | 0.434 | 2.303 |
| Surgical | 0.658 | 1.519 | 0.658 | 1.519 | 0.658 | 1.519 | 0.658 | 1.519 |
| Sepsis | 0.848 | 1.179 | 0.848 | 1.179 | 0.848 | 1.179 | 0.848 | 1.179 |
| Traumatic brain injury | 0.638 | 1.567 | 0.638 | 1.567 | 0.638 | 1.567 | 0.638 | 1.567 |
| SOFA score | 0.870 | 1.149 | 0.870 | 1.149 | 0.870 | 1.149 | 0.870 | 1.149 |

*Abbreviations: SOFA score, sequential organ failure assessment score; VIF, variance inflation factor.*

Table S3. Primary and secondary outcomes in the subgroup of patients with septic shock.

| Outcome | Total | Saline | Bicarbonate Ringer's | Odds Ratio | *P-value* | Adjusted Odds Ratio (95% CI)* | *P-value* |
| --- | --- | --- | --- | --- | --- | --- | --- |
|  | n=137 | n=87 | n=50 | (95% CI) |  |  |  |
| Primary outcome |  |  |  |  |  |  |  |
| Major adverse kidney event within 30 days - no. (%) | 99 (72.3) | 67 (77.0) | 32 (64.0) | 0.531 (0.247-1.139) | 0.104 | 0.465 (0.177-1.223) | 0.121 |
| Components of primary outcome |  |  |  |  |  |  |  |
| 30-day mortality - no. (%) | 46 (33.6) | 26 (29.9) | 20 (40.0) | 1.564 (0.755-3.241) | 0.229 | 1.649 (0.714-3.809) | 0.242 |
| New receipt of renal-replacement therapy - no. (%) | 80 (58.4) | 59 (67.8) | 21 (42.0) | 0.344 (0.167-0.706) | 0.004 | 0.260 (0.097-0.697) | 0.007 |
| Among survivors - no. (%) | 44/91 (48.4) | 37/61 (60.7) | 7/30 (23.3) | 0.197 (0.073-0.531) | 0.001 | 0.233 (0.065-0.831) | 0.025 |
| Final creatinine level ≥200% of baseline - no. (%) | 54 (39.4) | 33 (37.9) | 21 (42.0) | 1.185 (0.583-2.408) | 0.639 | 1.263 (0.514-3.108) | 0.611 |
| Among survivors - no. (%) | 32/91 (35.2) | 22/61 (36.1) | 10/30 (33.3) | 0.886 (0.353-2.228) | 0.798 | 2.029 (0.564-7.304) | 0.279 |
| Among survivors without new renal-replacement therapy - no. (%) | 9/47 (19.1) | 4/24 (16.7) | 5/23 (21.7) | 1.389 (0.322-5.986) | 0.659 | 1.855 (0.251-13.731) | 0.545 |
| Secondary outcomes |  |  |  |  |  |  |  |
| Hospital stay - day | 15.0 (6.0-28.0) | 15.0 (6.0-28.0) | 13.5 (4.0-27.3) |  | 0.537 |  |  |
| Length of ICU stay - day | 5.0 (3.0-9.0) | 5.0 (3.0-8.0) | 4.0 (2.0-9.0) |  | 0.244 |  |  |
| Vasopressor days - day | 2.0 (1.0-4.0) | 3.0 (1.0-5.0) | 2.0 (1.0-4.0) |  | 0.020 |  |  |
| New renal-replacement therapy days - day | 1.0 (0-4.0) | 2.0 (0-4.0) | 0 (0-2.3) |  | 0.005 |  |  |
| Among new renal-replacement therapy - day | 3.0 (2.0-5.0) | 3.0 (2.0-5.0) | 3.0 (1.5-4.0) |  | 0.760 |  |  |
| Mechanical ventilation days - day | 2.0 (0-4.5) | 2.0 (0-6.0) | 1.0 (0-3.0) |  | 0.101 |  |  |
| ICU-free days - day | 6.0 (0-16.0) | 6.0 (0-17.0) | 6.0 (0-15.0) |  | 0.886 |  |  |
| Ventilator-free days - day | 11.0 (1.0-23.0) | 11.0 (2.0-23.0) | 10.5 (1.0-25.0) |  | 0.884 |  |  |
| Vasopressor-free days - day | 11.0 (1.0-24.5) | 12.0 (2.0-24.0) | 10.5 (1.0-25.0) |  | 0.882 |  |  |
| New renal-replacement therapy-free days - day | 12.0 (2.5-27.0) | 11.0 (4.0-27.0) | 12.0 (1.8-27.0) |  | 0.971 |  |  |
| Among new renal-replacement therapy - day | 7.0 (1.0-16.0) | 8.0 (1.0-16.0) | 2.0 (0-19.0) |  | 0.286 |  |  |
| Cumulative fluid volume for resuscitation or maintenance within 24 hours - ml | 1000 (500-1000) | 500 (500-1000) | 1000 (500-1125) |  | 0.191 |  |  |
|  |  |  |  |  |  |  |  |

**Adjusted by age, gender, admission source, SOFA score at ICU admission and diagnosis on ICU admission, including medical diseases, cardiovascular disease, neurological disease, traumatic, surgical, sepsis and traumatic brain injury.*

*Quantitative variables are expressed as medians (interquartile ranges). Categorical variables were reported as the number of events (proportions).*

*Abbreviations: ICU, intensive care unit; SOFA, sequential organ failure assessment.*

| Table S4. Partial pressure of carbon dioxide in arterial blood gas analysis over time for bicarbonate Ringer’s group and saline group. | | | | |
| --- | --- | --- | --- | --- |
| Safety prognosis | Total | Saline | Bicarbonate Ringer's | *P* value |
| PaCO2 | n=662 | n=392 | n=270 |  |
| 0 hour PaCO2 - mmHg | 35.0 (30.0-41.0), n=661 | 35.0 (29.0-40.8), n=392 | 35.0 (30.0-41.0), n=269 | 0.409 |
| Chronic obstructive pulmonary disease - mmHg | 46.0 (38.5-66.5), n=13 | 48.5 (34.0-72.3), n=8 | 45.0 (38.5-63.0), n=5 | 0.558 |
| Without mechanical ventilation - mmHg | 34.0 (29.0-39.0), n=283 | 34.0 (29.0-39.8), n=172 | 34.0 (30.0-38.0), n=111 | 0.615 |
| With mechanical ventilation - mmHg | 36.0 (30.0-43.0), n=378 | 35.0 (29.0-42.0), n=220 | 37.0 (31.0-43.0), n=158 | 0.186 |
| 0 hour PaCO2＞50mmHg - no. (%) | 49/661 (7.4) | 28/392 (7.1) | 21/269 (7.8) | 0.749 |
| Chronic obstructive pulmonary disease - no. (%) | 4/13 (30.8) | 3/8 (37.5) | 1/5 (20.0) | 1.000 |
| Without mechanical ventilation - no. (%) | 8/283 (2.8) | 7/172 (4.1) | 1/111 (0.9) | 0.154 |
| With mechanical ventilation - no. (%) | 41/378 (10.8) | 21/220 (9.5) | 20/158 (12.7) | 0.337 |
| 6 hours PaCO2 - mmHg | 37.0 (32.0-42.0), n=485 | 37.0 (32.0-43.0), n=279 | 37.0 (31.0-41.0), n=206 | 0.087 |
| Chronic obstructive pulmonary disease - mmHg | 43.0 (36.5-51.0), n=12 | 43.0 (32.3-52.5), n=8 | 45.5 (38.5-51.0), n=4 | 0.933 |
| Without mechanical ventilation - mmHg | 37.0 (32.0-41.0), n=193 | 36.0 (32.0-41.0), n=111 | 37.0 (32.0-41.0), n=82 | 0.633 |
| With mechanical ventilation - mmHg | 38.0 (31.3-43.0), n=292 | 39.0 (32.0-45.0), n=168 | 37.0 (30.3-41.0), n=124 | 0.013 |
| 6 hours PaCO2＞50mmHg, no. (%) | 39/485 (8.0) | 28/279 (10.0) | 11/206 (5.3) | 0.060 |
| Chronic obstructive pulmonary disease - no. (%) | 5/12 (41.7) | 3/8 (37.5) | 2/4 (50.0) | 1.000 |
| Without mechanical ventilation - no. (%) | 8/193 (4.1) | 5/111 (4.5) | 3/82 (3.7) | 1.000 |
| With mechanical ventilation - no. (%) | 31/292 (10.6) | 23/168 (13.7) | 8/124 (6.5) | 0.047 |
| 0-6 hour PaCO2 change |  |  |  |  |
| 0-6 hour PaCO2 change value - mmHg | 2.0 (-3.0-6.0), n=484 | 2.0 (-3.0-7.0), n=279 | 0 (-4.0-5.0), n=205 | 0.017 |
| Chronic obstructive pulmonary disease - mmHg | -3.5 (-9.3-2.8), n=12 | -3.5 (-9.3-2.5), n=8 | -4.0 (-24.3-4.3), n=4 | 0.932 |
| Without mechanical ventilation - mmHg | 2.0 (-2.0-6.0), n=193 | 2.0 (-2.0-5.0), n=111 | 3.0 (-2.0-7.0), n=82 | 0.289 |
| With mechanical ventilation - mmHg | 1.0 (-4.0-6.0), n=291 | 3.0 (-3.0-7.8), n=168 | -1.0 (-7.0-4.0), n=123 | ＜0.001 |
| 0-6 hour PaCO2 increased - no. (%) | 271/484 (56.0) | 169/279 (60.6) | 102/205 (49.8) | 0.018 |
| Chronic obstructive pulmonary disease - no. (%) | 3/12 (25.0) | 2/8 (25.0) | 1/4 (25.0) | 1.000 |
| Without mechanical ventilation - no. (%) | 116/193 (60.1) | 66/111 (59.5) | 50/82 (61.0) | 0.832 |
| With mechanical ventilation - no. (%) | 155/291 (53.3) | 103/168 (61.3) | 52/123 (42.3) | 0.001 |
| Patients with elevated PaCO2 |  |  |  |  |
| 6 hours PaCO2 - mmHg | 38.0 (34.0-43.0), n=271 | 39.0 (34.0-43.0), n=169 | 38.0 (33.0-43.0), n=102 | 0.681 |
| Without mechanical ventilation - mmHg | 38.0 (35.0-43.0), n=116 | 37.5 (35.0-42.3), n=66 | 39.0 (34.5-43.3), n=50 | 0.543 |
| With mechanical ventilation - mmHg | 39.0 (33.0-44.0), n=155 | 40.0 (33.0-45.0), n=103 | 38.0 (33.0-42.8), n=52 | 0.384 |
| 0-6 hour PaCO2 increase value - mmHg | 6.0 (3.0-9.0), n=271 | 6.0 (3.0-9.0), n=169 | 5.0 (3.0-9.0), n=102 | 0.760 |
| Without mechanical ventilation - mmHg | 5.0 (3.0-8.0), n=116 | 4.5 (2.0-7.3), n=66 | 6.0 (3.0-9.3), n=50 | 0.085 |
| With mechanical ventilation - mmHg | 6.0 (3.0-10.0), n=155 | 7.0 (3.0-11.0), n=103 | 4.5 (3.0-8.0), n=52 | 0.093 |
| Patients without elevated PaCO2 |  |  |  |  |
| 6 hours PaCO2 - mmHg | 35.0 (30.0-41.0), n=213 | 36.0 (30.0-43.0), n=110 | 35.0 (30.0-39.0), n=103 | 0.265 |
| Without mechanical ventilation - mmHg | 35.0 (29.0-38.0), n=77 | 35.0 (28.5-39.0), n=45 | 34.0 (30.3-37.8), n=32 | 0.641 |
| With mechanical ventilation - mmHg | 36.5 (30.0-43.0), n=136 | 38.0 (31.0-45.0), n=65 | 35.0 (29.0-41.0), n=71 | 0.053 |
| 0-6 hour PaCO2 increase value - mmHg | -4.0 (-8.0--2.0), n=213 | -4.0 (-7.0--2.0), n=110) | -4.0 (-9.0--2.0), n=103 | 0.438 |
| Without mechanical ventilation - mmHg | -3.0 (-4.0--1.0), n=77 | -3.0 (-6.0--1.0), n=45 | -2.5 (-4.0--1.0), n=32 | 0.754 |
| With mechanical ventilation - mmHg | -5.0 (-10.0--3.0), n=136 | -5.0 (-9.5--2.5), n=65 | -6.0 (-11.0--3.0), n=71 | 0.425 |
| 24 hours PaCO2 - mmHg | 38.0 (32.0-43.0), n=640 | 38.0 (33.0-43.0), n=378 | 37.0 (32.0-42.0), n=262 | 0.140 |
| Chronic obstructive pulmonary disease - mmHg | 41.0 (36.0-60.0), n=13 | 41.0 (35.5-64.0), n=8 | 41.0 (37.0-57.0), n=5 | 0.942 |
| Without mechanical ventilation - mmHg | 37.0 (33.0-41.3), n=270 | 38.0 (33.0-41.0), n=163 | 37.0 (31.0-42.0), n=107 | 0.459 |
| With mechanical ventilation - mmHg | 38.0 (32.0-44.0), n=370 | 38.0 (32.0-45.0), n=215 | 37.0 (32.0-42.0), n=155 | 0.199 |
| 24 hours PaCO2＞50mmHg, no. (%) | 52/640 (8.1) | 31/378 (8.2) | 21/262 (8.0) | 0.933 |
| Chronic obstructive pulmonary disease - no. (%) | 5/13 (38.5) | 3/8 (37.5) | 2/5 (40.0) | 0.928 |
| Without mechanical ventilation - no. (%) | 7/270 (2.6) | 3/163 (1.8) | 4/107 (3.7) | 0.440 |
| With mechanical ventilation - no. (%) | 45/370 (12.2) | 28/215 (13.0) | 17/155 (11.0) | 0.551 |
| 6-24 hour PaCO2 change |  |  |  |  |
| 6-24 hour PaCO2 change value - mmHg | 1.0 (-3.0-6.0), n=463 | 1.0 (-3.5-6.0), n=265 | 1.5 (-3.0-6.3), n=198 | 0.537 |
| Chronic obstructive pulmonary disease - mmHg | 2.5 (-2.8-8.5), n=12 | 2.5 (-2.8-8.5), n=8 | 2.0 (-3.5-9.0), n=4 | 0.865 |
| Without mechanical ventilation - mmHg | 1.0 (-3.0-4.0), n=180 | 1.0 (-2.0-5.0), n=102 | 0 (-3.3-4.0), n=78 | 0.202 |
| With mechanical ventilation - mmHg | 2.0 (-4.0-7.0), n=283 | 1.0 (-4.0-6.0), n=163 | 3.0 (-3.0-7.0), n=120 | 0.137 |
| 6-24 hour PaCO2 increased - no. (%) | 259/463 (55.9) | 145/265 (54.7) | 114/198 (57.6) | 0.540 |
| Chronic obstructive pulmonary disease - no. (%) | 9/12 (75.0) | 6/8 (75.0) | 3/4 (75.0) | 1.000 |
| Without mechanical ventilation - no. (%) | 91/180 (50.6) | 54/102 (52.9) | 37/78 (47.4) | 0.464 |
| With mechanical ventilation - no. (%) | 168/283 (59.4) | 91/163 (55.8) | 77/120 (64.2) | 0.158 |
| Patients with elevated PaCO2 |  |  |  |  |
| 24 hours PaCO2 - mmHg | 41.0 (36.0-46.0), n=259 | 41.0 (36.0-46.0), n=145 | 40.0 (35.8-45.3), n=114 | 0.582 |
| Without mechanical ventilation - mmHg | 41.0 (36.0-44.0), n=91 | 40.5 (35.8-43.0), n=54 | 41.0 (36.0-46.5), n=37 | 0.190 |
| With mechanical ventilation - mmHg | 40.5 (35.3-48.8), n=168 | 43.0 (36.0-50.0), n=91 | 40.0 (35.0-45.0), n=77 | 0.166 |
| 6-24 hour PaCO2 increase value - mmHg | 5.0 (3.0-9.0), n=259 | 5.0 (2.0-9.0), n=145 | 5.0 (3.0-9.0), n=114 | 0.924 |
| Without mechanical ventilation - mmHg | 4.0 (2.0-8.0), n=91 | 4.0 (2.0-8.0), n=54 | 4.0 (1.0-8.0), n=37 | 0.600 |
| With mechanical ventilation - mmHg | 6.0 (3.0-10.0), n=168 | 5.0 (3.0-10.0), n=91 | 6.0 (3.0-10.0), n=77 | 0.793 |
| Patients without elevated PaCO2 |  |  |  |  |
| 24 hours PaCO2 - mmHg | 35.0 (30.0-39.0), n=204 | 36.0 (31.0-40.0), n=120 | 34.0 (29.3-38.0), n=84 | 0.010 |
| Without mechanical ventilation - mmHg | 35.0 (31.0-39.0), n=89 | 35.0 (33.0-39.0), n=48 | 34.0 (29.5-38.0), n=41 | 0.139 |
| With mechanical ventilation - mmHg | 34.0 (30.0-40.0), n=115 | 37.0 (30.0-41.0), n=72 | 33.0 (29.0-36.0), n=43 | 0.048 |
| 6-24 hour PaCO2 increase value - mmHg | -4.0 (-8.0--2.0), n=204 | -4.0 (-8.0--2.0), n=120 | -4.0 (-8.0--1.0), n=84 | 0.864 |
| Without mechanical ventilation - mmHg | -3.0 (-5.5--1.0), n=89 | -2.0 (-4.0--1.0), n=48 | -3.0 (-6.0--1.0), n=41 | 0.189 |
| With mechanical ventilation - mmHg | -5.0 (-11.0--3.0), n=115 | -5.0 (-11.0--3.0), n=72 | -5.0 (-9.0--1.0), n=43 | 0.514 |

*Continuous variables were presented as median (25th percentile - 75th percentile), n= total number. Categorical variables were reported as the number of events/total number (proportions).*

*Abbreviations: PaCO2, arterial partial pressure of carbon dioxide.*

| Table S5. PH in arterial blood gas analysis over time for bicarbonate Ringer’s group and saline group. | | | | |
| --- | --- | --- | --- | --- |
| Safety prognosis | Total | Saline | Bicarbonate Ringer's | *P-value* |
| pH | n=662 | n=392 | n=270 |  |
| 0 hour pH | 7.43 (7.36-7.48), n=661 | 7.43 (7.37-7.48), n=392 | 7.43 (7.36-7.49), n=269 | 0.484 |
| Chronic kidney disease | 7.41 (7.35-7.46), n=47 | 7.43 (7.37-7.47), n=26 | 7.38 (7.29-7.46), n=21 | 0.123 |
| Without new renal replacement therapy | 7.45 (7.39-7.49), n=452 | 7.44 (7.39-7.49), n=252 | 7.46 (7.39-7.50), n=200 | 0.173 |
| With new renal replacement therapy | 7.39 (7.32-7.45), n=209 | 7.40 (7.34-7.46), n=140 | 7.37 (7.27-7.42), n=69 | 0.020 |
| 0 hour pH＞7.45 - no. (%) | 258/661 (39.0) | 142/392 (36.2) | 116/269 (43.1) | 0.074 |
| Chronic kidney disease - no. (%) | 14/47 (29.8) | 9/26 (34.6) | 5/21 (23.8) | 0.421 |
| Without new renal replacement therapy - no. (%) | 208/452 (46.0) | 105/252 (41.7) | 103/200 (51.5) | 0.037 |
| With new renal replacement therapy - no. (%) | 50/209 (23.9) | 37/140 (26.4) | 13/69 (18.8) | 0.227 |
| 6 hours pH | 7.42 (7.37-7.47), n=485 | 7.42 (7.37-7.47), n=279 | 7.43 (7.38-7.48), n=206 | 0.128 |
| Chronic kidney disease | 7.41 (7.37-7.47), n=40 | 7.42 (7.38-7.47), n=22 | 7.39 (7.26-7.45), n=18 | 0.161 |
| Without new renal replacement therapy | 7.44 (7.40-7.49), n=300 | 7.44 (7.38-7.48), n=158 | 7.45 (7.41-7.50), n=142 | 0.036 |
| With new renal replacement therapy | 7.40 (7.33-7.45), n=185 | 7.40 (7.35-7.45), n=121 | 7.39 (7.30-7.43), n=64 | 0.081 |
| 6 hours pH＞7.45 - no. (%) | 171/485 (35.3) | 93/279 (33.3) | 78/206 (37.9) | 0.302 |
| Chronic kidney disease - no. (%) | 13/40 (32.5) | 9/22 (40.9) | 4/18 (22.2) | 0.209 |
| Without new renal replacement therapy - no. (%) | 131/300 (43.7) | 65/158 (41.1) | 66/142 (46.5) | 0.352 |
| With new renal replacement therapy - no. (%) | 40/185 (21.6) | 28/121 (23.1) | 12/64 (18.8) | 0.490 |
| 0-6 hour pH change |  |  |  |  |
| 0-6 hour pH change value | 0 (-0.04-0.06), n=484 | 0 (-0.05-0.05), n=279 | 0.01 (-0.03-0.07), n=205 | 0.028 |
| Chronic kidney disease | 0 (-0.03-0.06), n=40 | -0.01 (-0.03-0.05), n=22 | 0.01 (-0.04-0.08), n=18 | 0.643 |
| Without new renal replacement therapy | 0 (-0.04-0.05), n=299 | -0.01 (-0.05-0.04), n=158 | 0.01 (-0.03-0.06), n=141 | 0.105 |
| With new renal replacement therapy | 0.01 (-0.05-0.08), n=185 | 0 (-0.05-0.06), n=121 | 0.03 (-0.03-0.11), n=64 | 0.072 |
| 0-6 hour pH increased - no. (%) | 240/484 (49.6) | 128/279 (45.9) | 112/205 (54.6) | 0.057 |
| Chronic kidney disease - no. (%) | 18/40 (45.0) | 8/22 (36.4) | 10/18 (55.6) | 0.225 |
| Without new renal replacement therapy - no. (%) | 142/299 (47.5) | 70/158 (44.3) | 72/141 (51.1) | 0.243 |
| With new renal replacement therapy - no. (%) | 98/185 (53.0) | 58/121 (47.9) | 40/64 (62.5) | 0.059 |
| Patients with elevated pH |  |  |  |  |
| 6 hours pH | 7.44 (7.38-7.49), n=240 | 7.44 (7.39-7.48), n=128 | 7.44 (7.38-7.49), n=112 | 0.790 |
| Chronic kidney disease | 7.42 (7.37-7.47), n=18 | 7.47 (7.39-7.47), n=8 | 7.40 (7.32-7.45), n=10 | 0.073 |
| Without new renal replacement therapy | 7.46 (7.41-7.51), n=142 | 7.46 (7.41-7.50), n=70 | 7.46 (7.41-7.51), n=72 | 0.426 |
| With new renal replacement therapy | 7.41 (7.36-7.46), n=98 | 7.43 (7.38-7.47), n=58 | 7.38 (7.30-7.44), n=40 | 0.015 |
| 0-6 hour pH increase value | 0.06 (0.03-0.10), n=240 | 0.05 (0.02-0.09), n=128 | 0.06 (0.03-0.11), n=112 | 0.185 |
| Chronic kidney disease | 0.07 (0.03-0.16), n=18 | 0.07 (0.03-0.15), n=8 | 0.07 (0.03-0.22), n=10 | 0.964 |
| Without new renal replacement therapy | 0.05 (0.02-0.08), n=142 | 0.05 (0.02-0.07), n=70 | 0.06 (0.03-0.09), n=72 | 0.175 |
| With new renal replacement therapy | 0.07 (0.03-0.13), n=98 | 0.07 (0.03-0.11), n=58 | 0.09 (0.04-0.17), n=40 | 0.240 |
| Patients without elevated pH |  |  |  |  |
| 6 hours pH | 7.41 (7.36-7.46), n=244 | 7.41 (7.34-7.46), n=151 | 7.43 (7.37-7.48), n=93 | 0.034 |
| Chronic kidney disease | 7.40 (7.31-7.46), n=22 | 7.41 (7.36-7.46), n=14 | 7.39 (7.25-7.47), n=8 | 0.431 |
| Without new renal replacement therapy | 7.43 (7.38-7.47), n=157 | 7.42 (7.38-7.46), n=88 | 7.44 (7.39-7.49), n=69 | 0.053 |
| With new renal replacement therapy | 7.39 (7.31-7.42), n=87 | 7.38 (7.32-7.42), n=63 | 7.40 (7.27-7.43), n=24 | 0.669 |
| 0-6 hour pH increase value | -0.04 (-0.07--0.01), n=244 | -0.05 (-0.07--0.02), n=151 | -0.04 (-0.08--0.01), n=93 | 0.817 |
| Chronic kidney disease | -0.03 (-0.06--0.02), n=22 | -0.03 (-0.05--0.01), n=14 | -0.05 (-0.12--0.02), n=8 | 0.159 |
| Without new renal replacement therapy | -0.03 (-0.07--0.01), n=157 | -0.03 (-0.07--0.01), n=88 | -0.03 (-0.07--0.01), n=69 | 0.660 |
| With new renal replacement therapy | -0.05 (-0.08--0.02), n=87 | -0.05 (-0.08--0.02), n=63 | -0.07 (-0.14--0.01), n=24 | 0.321 |
| 24 hours pH | 7.45 (7.40-7.49), n=641 | 7.45 (7.40-7.49), n=378 | 7.46 (7.41-7.50), n=263 | 0.067 |
| Chronic kidney disease | 7.43 (7.38-7.48), n=46 | 7.43 (7.37-7.47), n=26 | 7.45 (7.40-7.50), n=20 | 0.399 |
| Without new renal replacement therapy | 7.46 (7.42-7.50), n=435 | 7.46 (7.42-7.49), n=240 | 7.46 (7.43-7.51), n=195 | 0.117 |
| With new renal replacement therapy | 7.42 (7.37-7.47), n=206 | 7.43 (7.37-7.47), n=138 | 7.42 (7.37-7.48), n=68 | 0.887 |
| 24 hours pH＞7.45 - no. (%) | 300/641 (46.8) | 167/378 (44.2) | 133/263 (50.6) | 0.111 |
| Chronic kidney disease - no. (%) | 18/46 (39.1) | 9/26 (34.6) | 9/20 (45.0) | 0.474 |
| Without new renal replacement therapy - no. (%) | 232/435 (53.3) | 121/240 (50.4) | 111/195 (56.9) | 0.176 |
| With new renal replacement therapy - no. (%) | 68/206 (33.0) | 46/138 (33.3) | 22/68 (32.4) | 0.888 |
| 6-24 hour pH change |  |  |  |  |
| 6-24 hour pH change value | 0.02 (-0.03-0.06), n=464 | 0.02 (-0.03-0.06), n=265 | 0.01 (-0.03-0.06), n=199 | 0.328 |
| Chronic kidney disease | 0 (-0.04-0.05), n=39 | -0.03 (-0.05-0.03), n=22 | 0.02 (-0.01-0.10), n=17 | 0.026 |
| Without new renal replacement therapy | 0.01 (-0.03-0.06), n=282 | 0.02 (-0.02-0.07), n=146 | 0.01 (-0.03-0.04), n=136 | 0.053 |
| With new renal replacement therapy | 0.02 (-0.03-0.07), n=182 | 0.02 (-0.03-0.06), n=119 | 0.02 (-0.03-0.11), n=63 | 0.305 |
| 6-24 hour pH increased - no. (%) | 263/464 (56.7) | 155/265 (58.5) | 108/199 (54.3) | 0.364 |
| Chronic kidney disease - no. (%) | 18/39 (46.2) | 7/22 (31.8) | 11/17 (64.7) | 0.041 |
| Without new renal replacement therapy - no. (%) | 159/282 (56.4) | 88/146 (60.3) | 71/136 (52.2) | 0.172 |
| With new renal replacement therapy - no. (%) | 104/182 (57.1) | 67/119 (56.3) | 37/63 (58.7) | 0.753 |
| Patients with elevated pH |  |  |  |  |
| 24 hours pH | 7.47 (7.43-7.51), n=263 | 7.46 (7.43-7.50), n=155 | 7.47 (7.43-7.52), n=108 | 0.338 |
| Chronic kidney disease | 7.45 (7.42-7.50), n=18 | 7.44 (7.37-7.51), n=7 | 7.45 (7.42-7.50), n=11 | 0.683 |
| Without new renal replacement therapy | 7.47 (7.44-7.52), n=159 | 7.47 (7.43-7.52), n=88 | 7.47 (7.44-7.52), n=71 | 0.332 |
| With new renal replacement therapy | 7.46 (7.40-7.49), n=104 | 7.46 (7.40-7.49), n=67 | 7.45 (7.41-7.49), n=37 | 0.940 |
| 6-24 hour pH increase value | 0.05 (0.03-0.10), n=263 | 0.05 (0.03-0.10), n=155 | 0.05 (0.02-0.11), n=108 | 0.533 |
| Chronic kidney disease | 0.05 (0.02-0.12), n=18 | 0.04 (0.02-0.06), n=7 | 0.05 (0.02-0.13), n=11 | 0.491 |
| Without new renal replacement therapy | 0.05 (0.02-0.09), n=159 | 0.06 (0.03-0.09), n=88 | 0.04 (0.02-0.08), n=71 | 0.071 |
| With new renal replacement therapy | 0.06 (0.03-0.13), n=104 | 0.05 (0.03-0.10), n=67 | 0.10 (0.04-0.16), n=37 | 0.072 |
| Patients without elevated pH |  |  |  |  |
| 24 hours pH | 7.42 (7.37-7.46), n=201 | 7.41 (7.36-7.45), n=110 | 7.43 (7.38-7.47), n=91 | 0.066 |
| Chronic kidney disease | 7.38 (7.28-7.43), n=21 | 7.39 (7.33-7.43), n=15 | 7.30 (7.12-7.49), n=6 | 0.507 |
| Without new renal replacement therapy | 7.44 (7.39-7.48), n=123 | 7.43 (7.38-7.48), n=58 | 7.44 (7.41-7.48), n=65 | 0.164 |
| With new renal replacement therapy | 7.39 (7.33-7.43), n=78 | 7.39 (7.33-7.43), n=52 | 7.38 (7.31-7.43), n=26 | 0.637 |
| 6-24 hour pH increase value | -0.03 (-0.06--0.02), n=201 | -0.03 (-0.06--0.02), n=110 | -0.03 (-0.07--0.01), n=91 | 0.818 |
| Chronic kidney disease | -0.04 (-0.09--0.02), n=21 | -0.04 (-0.09--0.03), n=15 | -0.03 (-0.11-0), n=6 | 0.456 |
| Without new renal replacement therapy | -0.03 (-0.07--0.01), n=123 | -0.03 (-0.07--0.01), n=58 | -0.03 (-0.07--0.01), n=65 | 0.988 |
| With new renal replacement therapy | -0.04 (-0.06--0.03), n=78 | -0.04 (-0.06--0.03), n=52 | -0.04 (-0.06--0.03), n=26 | 0.923 |

*Continuous variables were presented as median (25th percentile - 75th percentile), n= total number. Categorical variables were reported as the number of events/total number (proportions).*

| Table S6. Plasma concentration of bicarbonate in arterial blood gas analysis over time for bicarbonate Ringer’s group and saline group. | | | | |
| --- | --- | --- | --- | --- |
| Safety prognosis | Total | Saline | Bicarbonate Ringer's | *P* value |
| HCO3- | n=662 | n=392 | n=270 |  |
| 0 hour HCO3- - mmol/L | 23.4 (19.6-26.9), n=661 | 23.1 (19.6-26.5), n=391 | 24.0 (19.5-27.1), n=270 | 0.194 |
| Chronic kidney disease - mmol/L | 19.5 (16.1-23.5), n=47 | 19.9 (17.5-22.6), n=26 | 18.9 (14.4-24.2), n=21 | 0.542 |
| Without new renal replacement therapy - mmol/L | 24.3 (20.9-27.3), n=452 | 23.8 (21.1-27.2), n=251 | 24.7 (20.9-27.7), n=201 | 0.474 |
| With new renal replacement therapy - mmol/L | 21.1 (16.2-24.6), n=209 | 20.8 (16.3-24.8), n=140 | 21.2 (16.1-24.2), n=69 | 0.923 |
| 0 hour HCO3-＞27mmol/L - no. (%) | 153/661 (23.1) | 85/391 (21.7) | 68/270 (25.2) | 0.302 |
| Chronic kidney disease - no. (%) | 3/47 (6.4) | 1/26 (3.8) | 2/21 (9.5) | 0.579 |
| Without new renal replacement therapy - no. (%) | 125/452 (27.7) | 68/251 (27.1) | 57/201 (28.4) | 0.765 |
| With new renal replacement therapy - no. (%) | 28/209 (13.4) | 17/140 (12.1) | 11/69 (15.9) | 0.448 |
| 6 hours HCO3- - mmol/L | 24.2 (20.7-27.8), n=484 | 24.3 (20.7-27.9), n=278 | 24.2 (20.7-27.7), n=206 | 0.783 |
| Chronic kidney disease - mmol/L | 22.4 (19.3-26.2), n=40 | 24.5 (20.9-26.9). n=22 | 20.8 (14.2-23.9), n=18 | 0.043 |
| Without new renal replacement therapy - mmol/L | 25.1 (22.1-28.5), n=299 | 25.1 (22.3-28.8), n=157 | 25.1 (21.6-28.1), n=142 | 0.472 |
| With new renal replacement therapy - mmol/L | 22.6 (19.5-26.0), n=185 | 22.9 (19.6-26.4), n=121 | 22.5 (19.4-25.2), n=64 | 0.437 |
| 6 hours HCO3-＞27mmol/L - no. (%) | 145/484 (30.0) | 86/278 (30.9) | 59/206 (28.6) | 0.586 |
| Chronic kidney disease - no. (%) | 8/40 (20.0) | 5/22 (22.7) | 3/18 (16.7) | 0.709 |
| Without new renal replacement therapy - no. (%) | 112/299 (37.5) | 62/157 (39.5) | 50/142 (35.2) | 0.445 |
| With new renal replacement therapy - no. (%) | 33/185 (17.8) | 24/121 (19.8) | 9/64 (14.1) | 0.329 |
| 0-6 hours HCO3- change |  |  |  |  |
| 0-6 hours HCO3- change value - mmol/L | 1.1 (-1.3-3.5), n=484 | 1.3 (-1.3-3.8), n=278 | 0.9 (-1.3-3.0), n=206 | 0.354 |
| Chronic kidney disease - mmol/L | 1.9 (-1.8-5.8), n=40 | 3.2 (0.2-7.1), n=22 | 0 (-3.1-3.8), n=18 | 0.040 |
| Without new renal replacement therapy - mmol/L | 0.8 (-1.3-2.7), n=299 | 0.7 (-1.4-2.9), n=157 | 0.9 (-1.2-2.4), n=142 | 0.935 |
| With new renal replacement therapy - mmol/L | 1.6 (-1.5-4.8), n=185 | 1.7 (-1.2-5.4), n=121 | 1.2 (-1.8-4.3), n=64 | 0.261 |
| 0-6 hours HCO3- increased - no. (%) | 304/484 (62.8) | 173/278 (62.2) | 131/206 (63.6) | 0.759 |
| Chronic kidney disease - no. (%) | 26/40 (65.0) | 17/22 (77.3) | 9/18 (50.0) | 0.072 |
| Without new renal replacement therapy - no. (%) | 182/299 (60.9) | 92/157 (58.6) | 90/142 (63.4) | 0.398 |
| With new renal replacement therapy - no. (%) | 122/185 (65.9) | 81/121 (66.9) | 41/64 (64.1) | 0.694 |
| Patients with elevated HCO3- |  |  |  |  |
| 6 hours HCO3- - mmol/L | 24.8 (21.5-28.1), n=304 | 24.9 (21.6-28.2), n=173 | 24.5 (20.9-28.0), n=131 | 0.886 |
| Chronic kidney disease - mmol/L | 23.7 (20.8-26.9), n=26 | 24.7 (21.1-27.0), n=17 | 22.4 (19.1-26.1), n=9 | 0.269 |
| Without new renal replacement therapy - mmol/L | 26.0 (23.1-28.5), n=182 | 26.0 (23.4-28.5), n=92 | 26.0 (22.2-28.7), n=90 | 0.472 |
| With new renal replacement therapy - mmol/L | 23.2 (20.1-26.8), n=122 | 23.2 (19.9-27.2), n=81 | 23.2 (20.6-26.2), n=41 | 0.994 |
| 0-6 hours HCO3- increase value | 2.5 (1.2-4.8), n=304 | 2.9 (1.4-5.1), n=173 | 2.2 (1.0-4.4), n=131 | 0.050 |
| Chronic kidney disease - mmol/L | 4.0 (1.9-7.2), n=26 | 4.4 (2.2-7.4), n=17 | 3.5 (1.4-6.0), n=9 | 0.388 |
| Without new renal replacement therapy - mmol/L | 2.2 (1.0-4.0), n=182 | 2.2 (1.2-4.1), n=92 | 2.1 (1.0-3.8), n=90 | 0.435 |
| With new renal replacement therapy - mmol/L | 3.7 (1.6-6.9), n=122 | 4.0 (1.7-7.5),n=81 | 3.4 (1.4-5.0), n=41 | 0.188 |
| Patients without elevated HCO3- |  |  |  |  |
| 6 hours HCO3- - mmol/L | 23.6 (19.8-26.7), n=180 | 23.5 (19.6-27.1), n=105 | 23.7 (20.0-26.3), n=75 | 0.898 |
| Chronic kidney disease - mmol/L | 19.4 (12.3-24.0), n=14 | 21.3 (16.1-26.1), n=5 | 17.3 (11.3-22.9), n=9 | 0.257 |
| Without new renal replacement therapy - mmol/L | 24.3 (20.9-28.5), n=117 | 24.0 (20.4-30.4), n=65 | 24.5 (21.0-27.7), n=52 | 0.904 |
| With new renal replacement therapy - mmol/L | 21.0 (17.7-24.2), n=63 | 21.5 (18.1-24.9), n=40 | 20.1 (16.7-24.0), n=23 | 0.222 |
| 0-6 hours HCO3- increase value - mmol/L | -1.9 (-3.1--0.9), n=180 | -1.9 (-2.9--0.9), n=105 | -2.2 (-3.4--0.9), n=75 | 0.367 |
| Chronic kidney disease - mmol/L | -2.3 (-4.9--1.3), n=14 | -2.0 (-3.1--1.3), n=5 | -2.5 (-5.9--1.2), n=9 | 0.463 |
| Without new renal replacement therapy - mmol/L | -1.7 (-2.9--0.8), n=117 | -1.6 (-2.8--0.8), n=65 | -2.1 (-3.1--0.8), n=52 | 0.286 |
| With new renal replacement therapy - mmol/L | -2.1 (-3.9--1.2), n=63 | -2.1 (-3.6--1.2), n=40 | -2.2 (-5.5--1.4), n=23 | 0.617 |
| 24 hours HCO3- - mmol/L | 26.2 (22.6-29.1), n=640 | 26.0 (22.5-29.0), n=378 | 26.3 (22.6-29.3), n=262 | 0.564 |
| Chronic kidney disease - mmol/L | 24.2 (21.4-28.4), n=45 | 24.8 (22.8-29.5), n=26 | 21.5 (20.6-28.4), n=19 | 0.058 |
| Without new renal replacement therapy - mmol/L | 26.6 (23.1-29.9), n=435 | 26.4 (22.9-29.8), n=240 | 27.1 (23.5-29.9), n=195 | 0.451 |
| With new renal replacement therapy - mmol/L | 25.3 (21.4-28.3), n=205 | 25.5 (21.5-28.2), n=138 | 24.5 (21.1-28.3), n=67 | 0.321 |
| 24hours HCO3-＞27mmol/L - no. (%) | 275/640 (43.0) | 153/378 (40.5) | 122/262 (46.6) | 0.126 |
| Chronic kidney disease - no. (%) | 15/45 (33.3) | 9/26 (34.6) | 6/19 (31.6) | 0.831 |
| Without new renal replacement therapy - no. (%) | 205/435 (47.1) | 107/240 (44.6) | 98/195 (50.3) | 0.238 |
| With new renal replacement therapy - no. (%) | 70/205 (34.1) | 46/138 (33.3) | 24/67 (35.8) | 0.725 |
| 6-24 hours HCO3- change |  |  |  |  |
| 6-24 hours HCO3- change value - mmol/L | 1.5 (-0.9-4.2), n=462 | 1.6 (-0.9-4.5), n=264 | 1.4 (-1.0-4.2), n=198 | 0.531 |
| Chronic kidney disease - mmol/L | 1.2 (-0.7-4.7), n=38 | 1.2 (-0.5-4.4), n=22 | 1.7 (-1.8-5.7), n=16 | 0.701 |
| Without new renal replacement therapy - mmol/L | 1.2 (-0.8-3.6), n=281 | 1.4 (-0.8-3.6), n=145 | 1.2 (-0.8-3.5), n=136 | 0.918 |
| With new renal replacement therapy - mmol/L | 2.1 (-1.7-5.8), n=181 | 2.1 (-1.1-6.2), n=119 | 2.3 (-2.5-5.2), n=62 | 0.718 |
| 6-24 hours HCO3- increased - no. (%) | 309/462 (66.9) | 179/264 (67.8) | 130/198 (65.7) | 0.628 |
| Chronic kidney disease – no. (%) | 27/38 (71.1) | 16/22 (72.7) | 11/16 (68.8) | 0.790 |
| Without new renal replacement therapy - no. (%) | 185/281 (65.8) | 96/145 (66.2) | 89/136 (65.4) | 0.892 |
| With new renal replacement therapy - no. (%) | 124/181 (68.5) | 83/119 (69.7) | 41/62 (66.1) | 0.619 |
| Patients with elevated HCO3- |  |  |  |  |
| 24 hours HCO3- - mmol/L | 27.1 (23.8-30.2), n=309 | 26.9 (23.8-30.3), n=179 | 27.2 (23.8-29.9), n=130 | 1.000 |
| Chronic kidney disease - mmol/L | 24.3 (21.9-289), n=27 | 25.1 (22.7-30.8), n=16 | 21.9 (20.6-28.4), n=11 | 0.109 |
| Without new renal replacement therapy - mmol/L | 27.9 (24.5-30.6), n=185 | 27.9 (23.8-31.2), n=96 | 28.0 (24.8-30.5), n=89 | 0.800 |
| With new renal replacement therapy - mmol/L | 26.0 (22.9-28.9), n=124 | 26.0 (23.2-29.2), n=83 | 25.9 (22.5-28.7), n=41 | 0.579 |
| 6-24 hours HCO3- increase value - mmol/L | 3.1 (1.5-6.0), n=309 | 3.1 (1.5-6.2), n=179 | 3.2 (1.4-5.8), n=130 | 0.774 |
| Chronic kidney disease - mmol/L | 3.3 (0.9-5.4), n=27 | 3.1 (0.5-5.2), n=16 | 4.4 (1.0-11.3), n=11 | 0.324 |
| Without new renal replacement therapy - mmol/L | 2.7 (1.3-5.0), n=185 | 2.7 (1.4-4.8), n=96 | 2.7 (1.3-5.1), n=89 | 0.964 |
| With new renal replacement therapy - mmol/L | 3.9 (2.0-7.3), n=124 | 3.6 (1.9-7.1), n=83 | 4.0 (2.3-8.6), n=41 | 0.636 |
| Patients without elevated HCO3- |  |  |  |  |
| 24 hours HCO3- - mmol/L | 24.1 (20.9-27.8), n=153 | 24.2 (21.3-27.5), n=85 | 24.1 (20.4-28.0), n=68 | 0.993 |
| Chronic kidney disease - mmol/L | 23.8 (21.1-27.7), n=11 | 24.2 (21.3-26.2), n=6 | 21.3 (19.6-29.9), n=5 | 0.855 |
| Without new renal replacement therapy - mmol/L | 25.5 (21.5-29.0), n=96 | 26.0 (21.8-29.0), n=49 | 25.3 (21.1-29.1), n=47 | 0.820 |
| With new renal replacement therapy - mmol/L | 23.2 (20.1-25.4), n=57 | 23.2 (20.6-25.5), n=36 | 21.3 (19.6-25.8), n=21 | 0.591 |
| 6-24 hours HCO3- increase value | -2.1 (-3.5--1.0), n=153 | -1.9 (-3.6--1.0), n=85 | -2.2 (-3.5--0.9), n=68 | 0.697 |
| Chronic kidney disease - mmol/L | -2.1 (-5.1--1.0), n=11 | -2.8 (-7.7--0.6), n=6 | -2.1 (-3.8--1.6), n=5 | 1.000 |
| Without new renal replacement therapy - mmol/L | -1.7 (-3.0--0.7), n=96 | -1.5 (-3.1--0.8), n=49 | -2.0 (-2.6--0.7), n=47 | 0.933 |
| With new renal replacement therapy - mmol/L | -3.1 (-4.6--1.9), n=57 | -2.9 (-4.3--1.5), n=36 | -3.3 (-5.7--2.4), n=21 | 0.206 |

*Continuous variables were presented as median (25th percentile - 75th percentile), n= total number. Categorical variables were reported as the number of events/total number (proportions).*

*Abbreviations: HCO3-, bicarbonate.*

| Table S7. Base excess in arterial blood gas analysis over time for bicarbonate Ringer’s group and saline group. | | | | |
| --- | --- | --- | --- | --- |
| Safety prognosis | Total | Saline | Bicarbonate Ringer's | P-value |
| BE | n=662 | n=392 | n=270 |  |
| 0 hour BE - mmol/L | -0.6 (-4.7-3.0), n=659 | -0.9 (-4.6-2.7), n=391 | -0.2 (-4.8-3.6), n=268 | 0.213 |
| Chronic kidney disease - mmol/L | -4.5 (-8.3--0.7), n=47 | -4.0 (-6.3--1.6), n=26 | -5.6 (-10.6--0.6), n=21 | 0.416 |
| Without new renal replacement therapy - mmol/L | 0.6 (-3.2-3.7), n=451 | 0.5 (-3.4-3.4), n=251 | 0.9 (-3.2-4.3), n=200 | 0.362 |
| With new renal replacement therapy - mmol/L | -3.6 (-8.2-0.3), n=208 | -3.6 (-7.8-0.5), n=140 | -3.7 (-9.4--0.8), n=68 | 0.520 |
| 0 hour BE＞3mmol/L - no. (%) | 162/659 (24.6) | 88/391 (22.5) | 74/268 (27.6) | 0.135 |
| Chronic kidney disease - no. (%) | 7/47 (14.9) | 4/26 (15.4) | 3/21 (14.3) | 1.000 |
| Without new renal replacement therapy - no. (%) | 135/451 (29.9) | 69/251 (27.5) | 66/200 (33.0) | 0.204 |
| With new renal replacement therapy - no. (%) | 27/208 (13.0) | 19/140 (13.6) | 8/68 (11.8) | 0.716 |
| 6 hours BE - mmol/L | 0.1 (-3.6-3.8),n=484 | 0.4 (-3.7-3.6), n=278 | 0 (-3.3-4.1), n=206 | 0.872 |
| Chronic kidney disease - mmol/L | -1.8 (-5.1-2.3), n=40 | 0.6 (-3.6-2.4), n=22 | -3.7 (-12.5--0.3), n=18 | 0.069 |
| Without new renal replacement therapy - mmol/L | 1.2 (-2.0-4.6), n=299 | 1.2 (-1.9-4.5), n=157 | 1.1 (-2.0-5.0), n=142 | 0.996 |
| With new renal replacement therapy - mmol/L | -2.0 (-5.5-1.4), n=185 | -1.7 (-5.4-1.7), n=121 | -2.8 (-5.8-0), n=64 | 0.229 |
| 6 hours BE＞3mmol/L - no. (%) | 142/484 (29.3) | 80/278 (28.8) | 62/206 (30.1) | 0.752 |
| Chronic kidney disease - no. (%) | 7/40 (17.5) | 4/22 (18.2) | 3/18 (16.7) | 1.000 |
| Without new renal replacement therapy - no. (%) | 110/299 (36.8) | 58/157 (36.9) | 52/142 (36.6) | 0.954 |
| With new renal replacement therapy - no. (%) | 32/185 (17.3) | 22/121 (18.2) | 10/64 (15.6) | 0.662 |
| 0-6 hour BE change |  |  |  |  |
| 0-6 hour BE change value - mmol/L | 1.0 (-1.2-3.0), n=482 | 0.9 (-1.3-3.7), n=278 | 1.0 (-1.0-2.8), n=204 | 0.933 |
| Chronic kidney disease - mmol/L | 1.4 (-1.2-5.8), n=40 | 2.8 (-0.8-8.0), n=22 | 0.5 (-3.3-4.5), n=18 | 0.166 |
| Without new renal replacement therapy - mmol/L | 0.7 (-1.2-2.3), n=298 | 0.4 (-1.3-2.4), n=157 | 0.8 (-1.0-2.4), n=141 | 0.622 |
| With new renal replacement therapy - mmol/L | 1.4 (-1.3-5.0), n=184 | 1.4 (-1.4-5.6), n=121 | 1.3 (-1.0-4.6), n=63 | 0.756 |
| 0-6 hour BE increased - no. (%) | 291/482 (60.4) | 165/278 (59.4) | 126/204 (61.8) | 0.593 |
| Chronic kidney disease - no. (%) | 25/40 (62.5) | 16/22 (72.7) | 9/18 (50) | 0.140 |
| Without new renal replacement therapy - no. (%) | 172/298 (57.7) | 88/157 (56.1) | 84/141 (59.6) | 0.539 |
| With new renal replacement therapy - no. (%) | 119/184 (64.7) | 77/121 (63.6) | 42/63 (66.7) | 0.683 |
| Patients with elevated BE |  |  |  |  |
| 6 hours BE - mmol/L | 0.8 (-2.9-4.4), n=291 | 1.0 (-2.4-4.1), n=165 | 0.5 (-3.2-4.9), n=126 | 0.931 |
| Chronic kidney disease - mmol/L | 0.5 (-3.6-3.1), n=25 | 0.8 (-2.2-3.6), n=16 | -3.3 (-7.5-2.9), n=9 | 0.157 |
| Without new renal replacement therapy - mmol/L | 1.8 (-1.3-5.2), n=172 | 1.5 (-0.8-4.6), n=88 | 2.2 (-2.3-5.5), n=84 | 0.946 |
| With new renal replacement therapy - mmol/L | -0.7 (-4.5-2.5), n=119 | 0 (-4.4-3.3), n=77 | -1.5 (-4.7-1.1), n=42 | 0.278 |
| 0-6 hour BE increased value - mmol/L | 2.4 (1.2-5.2), n=291 | 2.6 (1.3-5.6), n=165 | 2.2 (1.1-4.6), n=126 | 0.190 |
| Chronic kidney disease - mmol/L | 4.4 (1.5-8.5), n=25 | 4.5 (1.7-8.6), n=16 | 4.3 (1.5-8.9), n=9 | 0.910 |
| Without new renal replacement therapy - mmol/L | 2.0 (1.0-3.8), n=172 | 2.1 (1.0-4.3), n=88 | 1.9 (1.0-3.1), n=84 | 0.630 |
| With new renal replacement therapy - mmol/L | 3.9 (1.4-7.6), n=119 | 4.0 (1.7-7.9), n=77 | 3.6 (1.3-6.5), n=42 | 0.494 |
| Patients without elevated BE |  |  |  |  |
| 6 hours BE - mmol/L | -0.8 (-5.1-2.4), n=191 | -1.3 (-5.5-2.5), n=113 | -0.3 (-4.9-2.5), n=78 | 0.499 |
| Chronic kidney disease - mmol/L | -5.0 (-12.4--1.0), n=15 | -4.3 (-8.2-1.4), n=6 | -5.1 (-14.7--1.2), n=9 | 0.480 |
| Without new renal replacement therapy - mmol/L | 0.5 (-2.2-3.9), n=126 | 0.5 (-3.5-4.0), n=69 | 0.5 (-1.6-3.7), n=57 | 0.846 |
| With new renal replacement therapy - mmol/L | -4.3 (-6.9--0.9), n=65 | -3.7 (-6.7--0.9), n=44 | -5.1 (-7.0--1.2), n=21 | 0.492 |
| 0-6 hour BE increased value - mmol/L | -1.8 (-3.2--0.7), n=191 | -1.8 (-3.2--0.8), n=113 | -1.9 (-3.2--0.7), n=78 | 0.747 |
| Chronic kidney disease - mmol/L | -2.0 (-5.5--0.9), n=15 | -1.8 (-3.4--1.1), n=6 | -2.5 (-6.7--0.8), n=9 | 0.637 |
| Without new renal replacement therapy - mmol/L | -1.6 (-2.6--0.7), n=126 | -1.6 (-2.8--0.7), n=69 | -1.6 (-2.5--0.6), n=57 | 0.514 |
| With new renal replacement therapy - mmol/L | -3.0 (-5.1--1.0), n=65 | -2.5 (-4.0--0.9), n=44 | -3.9 (-6.6--0.9), n=21 | 0.165 |
| 24 hours BE - mmol/L | 2.2 (-1.3-5.1), n=640 | 2.0 (-1.5-4.6), n=378 | 2.5 (-1.2-5.3), n=262 | 0.224 |
| Chronic kidney disease - mmol/L | -0.7 (-3.1-4.5), n=45 | 0.7 (-2.7-4.7), n=26 | -2.2 (-3.7-4.2), n=19 | 0.260 |
| Without new renal replacement therapy - mmol/L | 2.9 (-0.5-5.4), n=435 | 2.6 (-0.7-5.2), n=240 | 3.2 (-0.1-5.9), n=195 | 0.216 |
| With new renal replacement therapy - mmol/L | 0.8 (-3.2-3.7), n=205 | 1.1 (-3.1-3.6), n=138 | 0.4 (-3.4-3.9), n=67 | 0.468 |
| 24 hours BE＞3mmol/L - no. (%) | 278/640 (43.4) | 157/378 (41.5) | 121/262 (46.2) | 0.243 |
| Chronic kidney disease - no. (%) | 16/45 (35.6) | 9/26 (34.6) | 7/19 (36.8) | 0.878 |
| Without new renal replacement therapy - no. (%) | 214/435 (49.2) | 113/240 (47.1) | 101/195 (51.8) | 0.328 |
| With new renal replacement therapy - no. (%) | 64/205 (31.2) | 44/138 (31.9) | 20/67 (29.9) | 0.768 |
| 6-24 hour BE change |  |  |  |  |
| 6-24 hour BE change value - mmol/L | 1.6 (-0.9-4.1), n=462 | 1.8 (-0.7-4.3), n=264 | 1.2 (-1.1-3.9),n=198 | 0.247 |
| Chronic kidney disease - mmol/L | 2.0 (-0.9-4.4), n=38 | 0.8 (-0.5-4.2), n=22 | 2.7 (-1.0-5.6), n=16 | 0.408 |
| Without new renal replacement therapy - mmol/L | 1.2 (-0.8-3.5), n=281 | 1.5 (-0.5-3.6), n=145 | 1.0 (-1.1-3.4), n=136 | 0.195 |
| With new renal replacement therapy - mmol/L | 2.2 (-1.6-6.3), n=181 | 2.2 (-1.2-6.2), n=119 | 2.3 (-2.1-6.7), n=62 | 1.000 |
| 6-24 hour BE increased - no. (%) | 301/462 (65.2) | 179/264 (67.8) | 122/198 (61.6) | 0.167 |
| Chronic kidney disease - no. (%) | 24/38 (63.2) | 14/22 (63.6) | 10/16 (62.5) | 0.943 |
| Without new renal replacement therapy - no. (%) | 184/281 (65.5) | 101/145 (69.7) | 83/136 (61.0) | 0.129 |
| With new renal replacement therapy - no. (%) | 117/181 (64.6) | 78/119 (65.5) | 39/62 (62.9) | 0.724 |
| Patients with elevated BE |  |  |  |  |
| 24 hours BE - mmol/L | 3.1 (-0.2-6.1), n=301 | 3.1 (-0.3-6.4), n=179 | 3.5 (0.4-5.9), n=122 | 0.921 |
| Chronic kidney disease - mmol/L | -0.7 (-2.9-6.1), n=24 | 0.2 (-1.9-6.6), n=14 | -2.0 (-3.8-6.0), n=10 | 0.412 |
| Without new renal replacement therapy - mmol/L | 4.1 (0.6-6.3), n=184 | 4.0 (0-7.0), n=101 | 4.2 (1.2-5.9), n=83 | 0.778 |
| With new renal replacement therapy - mmol/L | 2.1 (-1.3-5.1), n=117 | 2.3 (-1.5-5.2), n=78 | 1.8 (-1.1-5.1), n=39 | 0.880 |
| 6-24 hour BE increased value - mmol/L | 3.3 (1.7-5.9), n=301 | 3.2 (1.7-6.3), n=179 | 3.4 (1.7-5.6), n=122 | 0.842 |
| Chronic kidney disease - mmol/L | 3.8 (2.2-5.7), n=24 | 3.4 (0.8-4.9), n=14 | 4.7 (2.8-10.0), n=10 | 0.128 |
| Without new renal replacement therapy - mmol/L | 2.7 (1.3-4.5), n=184 | 2.5 (1.3-4.5), n=101 | 2.9 (1.3-4.5), n=83 | 0.842 |
| With new renal replacement therapy - mmol/L | 4.4 (2.3-8.3), n=117 | 4.2 (2.2-8.1), n=78 | 5.1 (2.5-8.5), n=39 | 0.364 |
| Patients without elevated BE |  |  |  |  |
| 24 hours BE - mmol/L | 0.5 (-3.6-3.7), n=161 | 0.5 (-3.6-3.4), n=85 | 0.4 (-3.8-4.7), n=76 | 0.416 |
| Chronic kidney disease - mmol/L | -2.4 (-5.0-3.8), n=14 | -0.9 (-4.5-3.2), n=8 | -2.9 (-7.8-5.3), n=6 | 0.699 |
| Without new renal replacement therapy - mmol/L | 1.7 (-1.9-5.2), n=97 | 1.5 (-1.5-4.0), n=44 | 1.8 (-2.8-6.2), n=53 | 0.589 |
| With new renal replacement therapy - mmol/L | -2.7 (-4.9-0.9), n=64 | -2.7 (-4.4-1.0), n=41 | -2.2 (-6.1-0.7), n=23 | 0.580 |
| 6-24 hour BE increased value - mmol/L | -1.9 (-3.2--0.8), n=161 | -1.8 (-3.5--0.8), n=85 | -1.9 (-3.1--0.8), n=76 | 0.681 |
| Chronic kidney disease - mmol/L | -1.6 (-5.3--0.3), n=14 | -3.2 (-7.7--0.3), n=8 | -1.6 (-2.7--0.7), n=6 | 0.604 |
| Without new renal replacement therapy - mmol/L | -1.4 (-2.3--0.7), n=97 | -1.5 (-2.4--0.6), n=44 | -1.2 (-2.3--0.8), n=53 | 0.769 |
| With new renal replacement therapy - mmol/L | -2.6 (-4.3--1.2), n=64 | -2.4 (-4.1--1.1), n=41 | -3.2 (-5.2--1.8), n=23 | 0.405 |

*Continuous variables were presented as median (25th percentile - 75th percentile), n= total number. Categorical variables were reported as the number of events/total number (proportions).*

*Abbreviations: BE, base excess.*
